# Diagnostic Yield and Incremental Value of 16S rRNA Gene PCR and Sequencing on Excised Heart-Valve Tissue in Infective Endocarditis: A Systematic Review and Meta-Analysis

**DOI:** 10.3390/diagnostics16193125

**Published:** 2026-09-25

**Authors:** Florina Olivia Buzdug, Mirela Indrieș, Victor Alexandru, Claudia Elena Stanis, Felicia Manole, Sabina Florina Călugăr-Solea, Corina-Maria Mocan, Dumitru Adrian Buzdug, Alexandra Manuela Buzle, Andrei Nicolae Csep, Ioana Manuela Caraman, Felicia Liana Andronie-Cioara, Georgeta Pasca, Andrei Dăescu, Gheorghe Adrian Osiceanu, Alina Stanca Osiceanu, Marius Rus

**Affiliations:** 1 Doctoral School of Biomedical Sciences, University of Oradea, 1 University Street, 410087 Oradea, Romania; costea.florinaolivia@student.uoradea.ro (F.O.B.); alexandru.victor@student.uoradea.ro (V.A.); sabi_florina95@yahoo.com (S.F.C.-S.); buzle.alexandramanuela@student.uoradea.ro (A.M.B.); dr.manuelacaraman@gmail.com (I.M.C.); rusmarius@uoradea.ro (M.R.); 2 Department of Medical Disciplines, Faculty of Medicine and Pharmacy, University of Oradea, 410073 Oradea, Romania; fcioara@uoradea.ro (F.L.A.-C.); osiceanuadrian@uoradea.com (G.A.O.); osiceanualina@uoradea.com (A.S.O.); 3 Clinical County Emergency Hospital Bihor, 410169 Oradea, Romania; 4 Clinical Hospital of Infectious Diseases and Pneumophthisiology “Dr. Victor Babeș”, 300173 Timișoara, Romania; boticicorina@yahoo.com; 5 Discipline of Otorhinolaryngology (ENT), Department of Surgery, Faculty of Medicine and Pharmacy, University of Oradea, 1st December Square 10, 410073 Oradea, Romania; 6 Pelican Clinical Hospital, 410450 Oradea, Romania; mitik_buzdug@yahoo.com; 7 Doctoral School Department, “Victor Babes” University of Medicine and Pharmacy, 300041 Timisoara, Romania; andrei.daescu@umft.ro

**Keywords:** infective endocarditis, 16S rRNA, broad-range PCR, heart-valve tissue, blood-culture-negative endocarditis, molecular diagnosis, diagnostic yield, meta-analysis

## Abstract

**Background/Objectives:** Broad-range bacterial 16S rRNA gene PCR followed by sequencing may improve the etiological diagnosis of infective endocarditis (IE), particularly when conventional cultures are negative. This systematic review and meta-analysis evaluated the overall molecular detection yield and incremental value of valve-tissue 16S molecular testing in surgically managed IE. **Methods:** PubMed/MEDLINE, Scopus, Web of Science Core Collection, and Europe PMC were searched for Open Access studies published from 2016 to 2026. Eligible studies evaluated broad-range bacterial 16S PCR with sequence-based identification on excised native or prosthetic heart-valve tissue. Random-effects generalized linear mixed models were used to pool overall diagnostic yield, strict blood-culture-negative IE (BCNE) yield, and strict incremental etiologic yield beyond conventional microbiology. Diagnostic accuracy was summarized descriptively when complete 2 × 2 data were available. **Results:** Eight studies comprising 925 patients, surgical cases, or IE episodes contributed to the primary analysis, with 704 study-reported molecular-positive results. The pooled diagnostic yield was 75.9% (95% CI, 68.7–82.0%), with substantial heterogeneity (I^2^ = 81.8%) and a 95% prediction interval of 49.2–91.2%. In strict BCNE, the pooled yield was 53.7% (95% CI, 41.2–65.8%). The strict incremental etiologic yield was 8.8% (95% CI, 6.1–12.5%). Sensitivity analyses yielded similar estimates. **Conclusions:** Valve-tissue 16S PCR/sequencing can provide valuable etiological information, particularly in BCNE. However, the substantial between-study heterogeneity and wide prediction interval indicate that its expected molecular detection yield varies considerably across clinical settings and is influenced by patient selection, local diagnostic algorithms, laboratory methods, and reference standards.

## 1. Introduction

Infective endocarditis (IE) remains a severe and clinically heterogeneous infection involving native or prosthetic heart valves, intracardiac devices, or other endocardial structures. Despite advances in antimicrobial therapy, multimodality imaging, cardiac surgery, and multidisciplinary care, IE continues to be associated with substantial morbidity, mortality, and resource utilization. Accurate identification of the causative microorganism is central to patient management because it guides antimicrobial selection and treatment duration, supports diagnostic classification, and may influence perioperative and postoperative therapeutic decisions. Contemporary diagnostic frameworks integrate clinical, microbiological, imaging, surgical, and pathological findings. Importantly, the 2023 Duke–International Society for Cardiovascular Infectious Diseases criteria expanded the role of molecular diagnostics by recognizing microorganism identification through polymerase chain reaction (PCR), amplicon sequencing, or metagenomic sequencing performed on appropriate cardiac tissue as evidence supporting the pathological diagnosis of IE [1,2].

Globally, the estimated annual incidence of IE is approximately 13.8 cases per 100,000 population, while in-hospital mortality has remained largely unchanged over the past two decades, ranging from 15% to 30%. Blood-culture-negative infective endocarditis accounts for up to 20% of IE cases, although its frequency varies according to the epidemiological setting, previous antimicrobial exposure, and the diagnostic strategy used. Cardiac surgery is required in approximately one-half of patients during the active phase of IE. The principal indications are heart failure resulting from severe valvular dysfunction or destruction, uncontrolled infection, and prevention of septic embolism in patients with large or persistent vegetations [1,3].

Blood cultures remain the cornerstone of the etiological diagnosis of IE; however, they may remain negative because of antimicrobial administration before sampling, inadequate blood-culture collection, or infection caused by fastidious, intracellular, or non-cultivable microorganisms. Blood-culture-negative infective endocarditis (BCNE) is particularly challenging because failure to identify the pathogen may delay targeted therapy and lead to prolonged empirical antimicrobial treatment. Serological testing and organism-specific PCR can be highly informative for selected pathogens, including *Coxiella burnetii*, *Bartonella* spp., and *Tropheryma whipplei*, but their diagnostic scope is necessarily limited to the organisms being specifically investigated. In patients undergoing cardiac surgery, excised valve tissue provides an additional opportunity for etiological diagnosis. Nevertheless, conventional valve culture frequently has limited sensitivity, particularly after prolonged preoperative antimicrobial exposure, and may also produce clinically uncertain results because of perioperative or laboratory contamination [1,3].

Broad-range bacterial 16S rRNA gene PCR followed by sequence-based organism identification offers a culture-independent approach capable of detecting bacterial DNA directly from excised valve tissue. Because the assay does not depend on microbial viability, it may retain diagnostic value after antimicrobial treatment and may identify difficult-to-culture organisms in patients with negative blood and valve cultures. Multiple surgical cohorts have demonstrated that valve-tissue 16S testing can provide clinically relevant bacterial identifications, establish an etiology in BCNE, and occasionally clarify discordant or potentially contaminated conventional microbiological findings [4,5,6,7,8,9]. More recent approaches have extended molecular testing to targeted 16S next-generation sequencing and combined bacterial 16S/fungal 18S workflows, potentially increasing taxonomic breadth and permitting detection of mixed microbial signals [9,10,11]. However, molecular detection is not equivalent to demonstration of viable infection, and interpretation may be complicated by residual bacterial DNA, environmental or reagent contamination, sequence-quality thresholds, limited species-level resolution, and the frequent absence of phenotypic antimicrobial-susceptibility data.

Despite a growing body of evidence, the reported contribution of valve-tissue 16S testing varies substantially across studies. Published cohorts differ in patient selection, definitions of definite or possible IE, blood-culture status, reference standards, preoperative antimicrobial exposure, specimen processing, PCR targets, sequencing workflows, contamination controls, and units of analysis. Moreover, studies have not used a uniform definition of molecular “diagnostic benefit.” Some report overall molecular positivity, whereas others report clinically adjudicated etiological identification, molecular-only diagnoses, confirmation of conventional results, resolution of discordant findings, or changes in antimicrobial management. Pooling these non-equivalent outcomes without distinction may therefore obscure the specific clinical contribution of molecular testing.

Accordingly, this systematic review and meta-analysis aimed to estimate the overall molecular detection yield of broad-range bacterial 16S rRNA gene PCR followed by sequencing performed on surgically excised heart-valve tissue in patients with IE. Secondary objectives were to quantify diagnostic yield in strict BCNE, determine the strict incremental etiological yield beyond conventional microbiological testing, describe diagnostic accuracy where complete 2 × 2 data were available, and characterize broader diagnostic and therapeutic contributions that could not be combined quantitatively.

## 2. Methods

### 2.1. Study Design and Reporting Framework

This systematic review and meta-analysis evaluated the diagnostic contribution of broad-range bacterial 16S rRNA/rDNA polymerase chain reaction (PCR) followed by sequencing performed on surgically excised heart-valve tissue in patients with infective endocarditis (IE).

A protocol describing the methodological and analytic framework of this systematic review and meta-analysis is available on protocols.io. The protocol for this systematic review and meta-analysis has been deposited on protocols.io (https://dx.doi.org/10.17504/protocols.io.e6nvwxj5zgmk/v1). The review was reported in accordance with the Preferred Reporting Items for Systematic Reviews and Meta-Analyses (PRISMA 2020) recommendations. An initial protocol specifying the eligibility criteria, outcome domains, and statistical framework was prepared before the literature search and study screening. It was formally registered after screening, data extraction, and statistical analyses had been conducted and is therefore described as retrospectively registered. Subsequent changes were limited to clarifying the operational definitions of the reported outcomes and did not affect study eligibility or the primary analytical framework.

The primary objective was to estimate the overall molecular detection yield of valve-tissue 16S molecular testing in patients or infective-endocarditis episodes undergoing surgical valve sampling. Secondary objectives were to estimate molecular diagnostic yield in blood-culture-negative infective endocarditis (BCNE), quantify the strict incremental etiologic yield beyond conventional microbiological testing, describe diagnostic accuracy where complete 2 × 2 data were available, and characterize additional molecular diagnostic benefit in selected culture-negative populations.

### 2.2. Eligibility Criteria

Studies were eligible if they reported original patient- or episode-level data from patients with definite, possible, suspected, or clinically adjudicated IE undergoing cardiac surgery with analysis of excised native or prosthetic heart-valve tissue using broad-range bacterial 16S rRNA gene PCR followed by sequence-based organism identification.

Eligible index tests included broad-range bacterial 16S rRN16S rRNA gene PCRA/rDNA PCR followed by Sanger sequencing, amplicon sequencing, or another sequence-based identification workflow. The primary quantitative synthesis was restricted to studies providing a clinically interpretable bacterial 16S result at the patient or IE-episode level and an extractable numerator and denominator for diagnostic yield.

Clinical diagnosis of IE was accepted according to the study-specific reference standard, including modified Duke criteria, contemporary guideline-based clinical adjudication, postoperative classification, histopathology, operative findings, or a composite clinical/pathological reference standard.

Studies were excluded from the primary quantitative synthesis when they consisted exclusively of case reports, single-patient descriptions, reviews, conference abstracts without sufficient cohort-level data, pathogen-specific PCR studies, studies without excised valve-tissue testing, studies lacking an interpretable IE target-condition reference standard, or reports from which patient- or episode-level diagnostic yield could not be determined.

Studies using targeted 16S next-generation sequencing with predominantly specimen-level outcomes were retained for exploratory synthesis but were not pooled with the primary patient/episode-level PCR/sequencing studies. Similarly, studies reporting aggregate bacterial 16S and fungal 18S molecular results without separable bacterial counts were retained for secondary or descriptive analyses but excluded from the strict bacterial 16S primary meta-analysis.

### 2.3. Information Sources and Search Strategy

A systematic literature search was conducted in PubMed/MEDLINE, Scopus, Web of Science Core Collection, and Europe PMC, with the final searches performed in July 2026.

The search strategy combined three conceptual groups of terms:

1. Infective-endocarditis terminology, including infective endocarditis, infectious endocarditis, bacterial endocarditis, blood culture-negative endocarditis, culture-negative endocarditis, native-valve endocarditis, and prosthetic-valve endocarditis;
2. Molecular-diagnostic terminology, including 16S rRNA, 16S rDNA, 16S ribosomal RNA, 16S ribosomal DNA, 16S sequencing, 16S amplicon sequencing, broad-range PCR, broad-range bacterial PCR, panbacterial PCR, universal bacterial PCR, and related terms;
3. Surgical-specimen terminology referring to heart valves, excised valve tissue, resected valves, cardiac valve tissue, or valvular vegetations.

Database-specific syntax was adapted to the indexing structure of each database. Boolean operators and field-specific search functions were used where supported.

Searches were restricted to publications from 2016 through 2026 and to records available under the respective database Open Access filters. Reference lists and bibliographic information from relevant full-text publications were additionally examined to identify potentially overlapping reports and related cohorts. The final searches were performed in July 2026. The complete database-specific search strategies, including all Boolean operators, field restrictions, publication-period limits, and Open Access filters, are available in the registered protocols.io document.

The publication-period and Open Access restrictions were defined as scope criteria and applied consistently across all database searches. The 2016 publication cutoff was selected to focus the review on contemporary clinical and laboratory practice, given the evolution of IE diagnostic criteria, specimen-processing procedures, amplification targets, sequencing workflows, contamination-control measures, and the clinical interpretation of valve-tissue molecular results. The Open Access restriction was used to ensure that the complete reports underlying the extracted numerators, denominators, assay characteristics, and outcome definitions were directly accessible and independently verifiable. Open Access status was not considered an indicator of methodological quality; the quality and risk of bias of the included studies were evaluated separately using the adapted QUADAS-2 framework. These restrictions therefore delimit the intended scope of the review to contemporary, openly accessible evidence and do not imply that earlier or non-Open Access studies are intrinsically less relevant or methodologically inferior.

### 2.4. Record Management, Deduplication, and Study Selection

Search results from the four databases were combined into a single screening dataset. Duplicate records were identified using available bibliographic identifiers, including DOI and PMID, supplemented by normalized title, author, publication year, and bibliographic matching when unique identifiers were unavailable or inconsistent. Potential duplicate groups were reviewed to distinguish true duplicate database records from separate publications originating from the same or overlapping study populations.

Study selection was performed in two stages. First, titles and abstracts were screened against the predefined eligibility criteria. Records considered potentially eligible proceeded to full-text assessment. Second, full-text articles were evaluated for target population, specimen type, index test, reference standard, availability of cohort-level diagnostic data, and suitability for one or more quantitative or descriptive outcomes. Reasons for exclusion at full-text assessment were recorded. Two reviewers independently screened titles and abstracts and assessed potentially eligible full-text reports. Disagreements were resolved by consensus.

**Figure 1:**
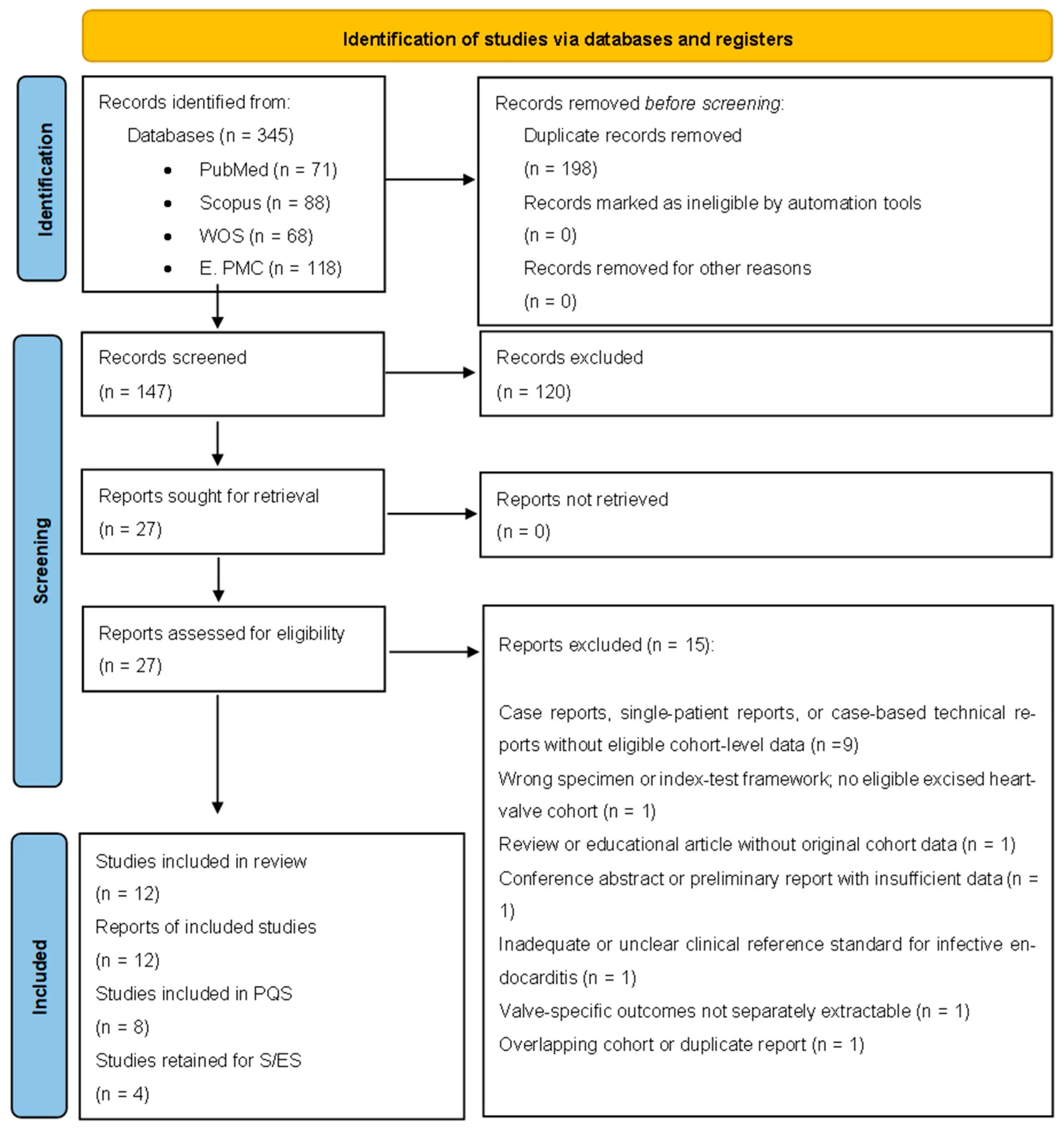
PRISMA 2020 flow diagram of study identification, screening, eligibility assessment, and inclusion. Records retrieved from PubMed/MEDLINE, Scopus, Web of Science Core Collection, and Europe PMC were combined and deduplicated before title/abstract screening and full-text eligibility assessment. Reasons for exclusion at the full-text stage are shown. Studies retained for the systematic review but not eligible for the primary quantitative synthesis were included in secondary or exploratory analyses where appropriate. Full-text exclusions included reports with insufficient cohort-level data, an ineligible specimen or index-test framework, an inadequate clinical reference standard, outcomes that were not separately extractable, and an overlapping cohort. Abbreviation: PRISMA, Preferred Reporting Items for Systematic Reviews and Meta-Analyses.

The complete study-selection process, from database identification through deduplication, title/abstract screening, full-text assessment, and final inclusion, is presented in the PRISMA 2020 flow diagram (Figure 1).

### 2.5. Management of Overlapping Cohorts

When multiple publications were identified as arising from the same or substantially overlapping patient population, cohort overlap was explicitly assessed using recruitment center, enrollment period, sample size, and study characteristics.

To avoid double-counting participants, only one report from an overlapping cohort was used for a given pooled outcome. The report providing the most complete or appropriate data for the prespecified endpoint was prioritized. Secondary publications from overlapping cohorts could be retained for qualitative synthesis or for a different outcome if they provided non-duplicative information not available from the report selected for the primary analysis.

### 2.6. Data Extraction and Full-Text Verification

Data were extracted into a structured study-level database. Extracted variables included study identification, country, study design, recruitment period, number of centers, population definition, reference standard, unit of analysis, number of enrolled patients or IE episodes, number of valve specimens, specimen type and preservation, molecular assay, PCR target, sequencing method, preoperative antimicrobial exposure where available, and results of conventional and molecular microbiological testing. Two reviewers independently extracted the study characteristics and outcome data. Disagreements were resolved by consensus. Detailed methodological and population characteristics of the included studies are provided in Table S1.

For each study included in the primary meta-analysis, we additionally extracted the targeted 16S region and primers, sequencing method and platform, sequence-quality and taxonomic-similarity criteria, approach to mixed sequences, and reference database. When a primary report referred explicitly to a previously published assay protocol, information available in that cited protocol was also considered. Variables that could not be verified from the published reports were recorded as not reported. These study-level laboratory characteristics are presented in Table S12.

Outcome-specific fields included the number of molecular tests performed, study-reported molecular-positive results, reported taxonomic resolution, results classified by the original authors as contaminants or uninterpretable, blood-culture and valve-culture results, BCNE denominators and molecular-positive counts, cases with both blood and valve cultures negative, study-defined diagnostic benefit, strict incremental etiologic benefit, management changes, and TP, FP, FN, and TN counts where reconstructable.

All studies contributing to the quantitative analyses underwent full-text re-verification of numerators, denominators, endpoint definitions, and analysis units. We did not retrospectively impose a uniform organism-level clinical adjudication that was unavailable in the original reports. Results explicitly classified by the original investigators as contaminants or non-interpretable were excluded when this distinction was reported. Otherwise, the study-reported molecular-positive count was retained. Study-specific handling of potentially ambiguous molecular findings is summarized in Table S11.

When discrepant values were reported within the same publication, we prioritized values presented in the full-text Results and corresponding tables or figures over those reported in the Abstract. When discrepancies occurred among full-text sources, we selected the value that most closely matched the prespecified outcome definition, eligible population, and unit of analysis. Where more than one value remained plausible, the alternative value was examined in a sensitivity analysis and the discrepancy was reported explicitly.

### 2.7. Outcome Definitions

#### 2.7.1. Primary Molecular Detection Yield

The primary outcome was the overall molecular detection yield of valve-tissue bacterial 16S PCR followed by sequencing. It was defined as the proportion of eligible patients or infective-endocarditis episodes with a molecular-positive result, as reported in the original study, among those undergoing valve-tissue 16S testing.

This outcome quantifies study-reported molecular detection and should not be interpreted as uniform patient-level proof of etiological causation or clinical benefit. Results explicitly classified by the original investigators as contaminants, failed sequences, or non-interpretable findings were excluded when such information was available. Genus-level or discordant findings were retained when they were included in the molecular-positive count of the original study. Clinically incremental etiologic information was evaluated separately through the strict incremental etiologic yield and broad diagnostic-benefit outcomes. Study-level definitions and the handling of potentially ambiguous results are provided in Table S11.

#### 2.7.2. Blood-Culture-Negative Infective Endocarditis

The strict BCNE diagnostic yield was defined as the proportion of patients or IE episodes with negative blood cultures in whom valve-tissue 16S testing provided a study-reported molecular-positive result. Patients for whom blood cultures were unavailable were not included in the strict BCNE denominator. Studies combining blood-culture-negative cases with cases in which blood cultures were unavailable were therefore excluded from the strict BCNE pooled analysis and retained only for descriptive or sensitivity purposes. Here, “strict” refers specifically to documented blood-culture negativity rather than unavailable blood-culture results. Eligibility for this analysis did not require negative valve cultures, serology, or targeted molecular assays, or documented completion of a comprehensive diagnostic work-up. Thus, this endpoint measures molecular detection among blood-culture-negative cases, not necessarily new etiological identification after all other diagnostic investigations were unrevealing.

#### 2.7.3. Strict Incremental Etiologic Yield

The strict incremental etiologic yield was defined as the proportion of the eligible study population in whom bacterial 16S testing represented the sole positive or sole etiologically informative diagnostic method beyond conventional microbiological testing. This endpoint was deliberately distinguished from broader study-defined concepts of “diagnostic benefit.” Accordingly, cases in which molecular testing merely confirmed an already available conventional result, clarified a probable contaminant without being the sole etiologically informative method, or contributed to diagnostic exclusion were not automatically classified as strict incremental etiologic events. “Conventional microbiological testing” refers to the study-specific non-index microbiological work-up documented in the original reports, including blood and valve cultures and, where reported, serology and organism-specific molecular assays. This term does not imply that all patients underwent the same diagnostic panel. Incremental yield was therefore assessed against the documented study-specific comparator, rather than against a uniform comprehensive diagnostic algorithm.

#### 2.7.4. Broad Diagnostic Benefit

A separate broad diagnostic-benefit endpoint retained the study authors’ wider definitions of clinical or diagnostic contribution. Depending on the original study, this could include molecular-only identification, confirmation or clarification of uncertain conventional microbiology, resolution of discordant results, or contribution to diagnostic exclusion. Because these definitions were not sufficiently homogeneous across studies, broad diagnostic benefit was summarized descriptively and was not pooled as the strict incremental etiologic endpoint.

#### 2.7.5. Cases with Both Negative Blood and Valve Cultures

A further exploratory endpoint assessed molecular diagnostic yield among patients in whom both blood cultures and valve cultures were negative. Because the included studies differed in the precise definition and ascertainment of this subgroup, study-specific proportions and exact confidence intervals were presented descriptively rather than combined into a pooled summary estimate.

#### 2.7.6. Diagnostic Accuracy Assessment

Diagnostic accuracy was evaluated when a complete strict bacterial 16S 2 × 2 contingency table could be reconstructed against an acceptable study-specific reference standard. For each eligible study, sensitivity, specificity, positive predictive value (PPV), and negative predictive value (NPV) were calculated from TP, FP, FN, and TN counts. Studies using aggregate bacterial 16S/fungal 18S assays were not combined with the strict bacterial 16S accuracy analysis. Because only two studies provided suitable strict 16S 2 × 2 data, no pooled sensitivity, pooled specificity, bivariate diagnostic-accuracy model, or hierarchical summary receiver operating characteristic analysis was performed.

#### 2.7.7. Impact on Antimicrobial Management

Changes in antimicrobial treatment attributed to molecular valve testing were extracted where reported. Because management-impact definitions were sparse and heterogeneous, these results were retained as descriptive secondary outcomes and were not quantitatively pooled.

### 2.8. Risk-of-Bias Assessment

Risk of bias was evaluated using an adapted QUADAS-2 framework appropriate for studies of molecular diagnostic testing in infective endocarditis. Risk-of-bias judgments were independently performed by two reviewers, with disagreements resolved by consensus.

Four domains were assessed:

1. Patient selection;
2. Index test;
3. Reference standard;
4. Flow and timing.

Each domain was classified as low, unclear, or high risk of bias on the basis of the information provided in the full-text publication. Overall risk of bias was classified as high when at least one domain was judged high risk; studies without a high-risk domain but with one or more unclear domains were classified as overall unclear. Domain-level judgments and their supporting rationale were retained for supplementary reporting. A sensitivity analysis excluding studies classified as having high overall risk of bias was performed to evaluate the robustness of the primary pooled diagnostic-yield estimate.

### 2.9. Statistical Analysis

Statistical analyses were performed in R, version 4.3.0 (R Core Team, R Foundation for Statistical Computing, Vienna, Austria; 2024) using the meta and metafor packages. Data processing and coding were conducted in RStudio, version 2023.06.0 + 421 (Posit Software, PBC, Boston, MA, USA, 2023), an integrated development environment for R. Statistical significance was set at *p* < 0.05, two-tailed. For proportional outcomes, study-specific proportions were calculated from the extracted event and denominator counts. Exact binomial 95% confidence intervals were calculated for descriptive study-level estimates. Because overall molecular detection yield was expected to vary across populations, laboratory workflows, reference standards, and clinical settings, a random-effects framework was used for all pooled proportional outcomes.

#### 2.9.1. Primary Meta-Analysis

The primary diagnostic-yield meta-analysis was fitted using a random-intercept logistic generalized linear mixed model (GLMM) with logit transformation. Between-study variance (τ^2^) was estimated by maximum likelihood. Pooled estimates were back-transformed to proportions and reported with 95% confidence intervals. A 95% prediction interval was additionally calculated to estimate the range of underlying diagnostic yields that could plausibly be expected in a comparable future study. Prediction intervals were calculated using a t-distribution with k−1 degrees of freedom.

Between-study heterogeneity was quantified using:

- Cochran’s Q;
- I^2^;
- τ^2^;
- and τ.

A two-sided heterogeneity test *p* < 0.05 was considered statistically significant. The statistical specification used in the final analysis corresponded to a random-intercept logistic model with maximum-likelihood estimation of τ^2^ and logit transformation.

#### 2.9.2. Alternative Random-Effects Estimator

To assess sensitivity of the primary result to the statistical estimator, the primary analysis was repeated using an inverse-variance random-effects model with restricted maximum-likelihood (REML) estimation of between-study variance. Confidence intervals for τ^2^ and τ were obtained using the Q-profile approach. Agreement between the GLMM and inverse-variance REML estimates was used as a robustness check rather than as a separate primary analysis.

Because the GLMM does not yield conventional inverse-variance study weights, study weights displayed in the primary forest plot were derived from the corresponding inverse-variance REML model, while the pooled diamond and prediction interval represented the prespecified GLMM estimate.

#### 2.9.3. Leave-One-Out and Influence Analyses

Robustness of the primary meta-analysis was further assessed by sequentially excluding each study and refitting the random-effects model. For each leave-one-out model, pooled diagnostic yield, 95% confidence interval, τ^2^, and I^2^ were recorded. Formal influence diagnostics were additionally performed on logit-transformed proportions using a REML random-effects model in metafor.

Influence measures included:

- Studentized residuals;
- DFFITS;
- Cook’s distance;
- Covariance ratios;
- τ^2^\tau^2τ^2^ after study deletion;
- Residual heterogeneity after study deletion;
- Hat values;
- Study weights;
- And DFBETAS.

Influence analyses were used to identify studies contributing disproportionately to the pooled estimate or heterogeneity; influential observations were not automatically excluded from the primary model.

#### 2.9.4. Sensitivity Analyses

Several targeted sensitivity analyses were performed. First, because Kim et al. reported 43/79 molecular-positive cases in the Results/Figure but 40/79 in the abstract, the primary model used the full-text Results/Figure value of 43/79 and was repeated using 40/79 to quantify the effect of this within-publication discrepancy. Second, the primary meta-analysis was repeated after excluding studies with important methodological or denominator concerns individually and in combination. Third, a risk-of-bias sensitivity analysis excluded all studies classified as high overall risk of bias. For the strict incremental etiologic-yield model, additional sensitivity analyses were performed after excluding Peeters et al., Rodríguez-García et al., and both studies simultaneously to assess the effect of study-specific denominator or selection considerations.

#### 2.9.5. Secondary Pooled Outcomes

The strict BCNE diagnostic yield and strict incremental etiologic yield were analyzed using the same random-intercept logistic GLMM framework as the primary outcome. For each secondary pooled outcome, pooled proportions, 95% confidence intervals, 95% prediction intervals, τ^2^, I^2^, and Cochran’s Q were reported. The final strict BCNE and strict incremental models were constructed from outcome-specific datasets that were analytically separated from non-strict or broader diagnostic-benefit definitions. Exploratory assessments of funnel-plot asymmetry and small-study effects are described in the Supplementary Material.

#### 2.9.6. Subgroup Analysis and Meta-Regression

Potential study-level moderators were systematically reviewed before formal subgroup analysis or meta-regression. Candidate variables included country, study design, center structure, analysis unit, population definition, reference standard, specimen characteristics, PCR target, sequencing method, and other methodological characteristics. Formal subgroup analyses or meta-regression were not performed when a variable lacked sufficient replication across studies, produced groups containing only one or very few studies, represented outcome availability rather than a meaningful clinical or methodological characteristic, or could not support a statistically reliable between-group comparison. Given the small number of studies in the primary model and the sparse distribution of potentially relevant study-level characteristics, no clinically meaningful moderator met the requirements for reliable meta-regression.

### 2.10. Presentation of Results

For pooled proportional outcomes, results were presented as percentages with 95% confidence intervals and 95% prediction intervals. Forest plots displayed study-level estimates and exact 95% confidence intervals together with the random-effects summary estimate where pooling was considered appropriate. Diagnostic-accuracy results were presented separately for sensitivity and specificity without a pooled estimate. Outcomes considered insufficiently homogeneous for quantitative pooling—including broad study-defined diagnostic benefit and molecular yield after both negative blood and valve cultures—were summarized using study-level proportions and exact binomial confidence intervals. All statistical tests were two-sided.

## 3. Results

### 3.1. Study Characteristics

The database searches identified 345 records: 71 from PubMed/MEDLINE, 88 from Scopus, 68 from Web of Science Core Collection, and 118 from Europe PMC. After removal of 198 duplicate records, 147 unique records were screened by title and abstract, of which 120 were excluded. Twenty-seven reports underwent full-text assessment, and 15 were excluded for the reasons presented in Figure 1. Twelve studies were included in the review: eight contributed to the primary quantitative synthesis, while four were retained for secondary or exploratory synthesis. Eight studies provided patient- or episode-level data suitable for the primary quantitative synthesis of the diagnostic yield of broad-range bacterial 16S rRNA gene PCR followed by sequencing on excised heart-valve tissue. The studies were conducted in eight countries and included both prospective and retrospective surgical cohorts. The unit of analysis varied across studies and included individual patients, infective-endocarditis episodes, and surgical cases. Molecular workflows consisted predominantly of broad-range 16S PCR followed by Sanger sequencing or other sequence-based identification approaches.

A total of 925 patients or infective-endocarditis episodes contributed to the primary analysis, of whom 704 had a study-reported molecular-positive result. Individual study sample sizes ranged from 40 to 279, while study-specific diagnostic yields ranged from 54.4% in Kim et al. to 87.5% in Peeters et al. Seven of the eight studies reported diagnostic yields exceeding approximately two-thirds of the analyzed population. Detailed study characteristics, population definitions, reference standards, and molecular methods are presented in Table 1, with additional methodological details provided in Table S1. The operational definition of molecular positivity and the reported handling of contaminants, unresolved sequences, genus-level identifications, discordant findings, and potentially persistent bacterial DNA are summarized for each study in Table S11. Detailed study-level information regarding the targeted 16S regions and primers, sequencing platforms, sequence-quality and similarity criteria, handling of mixed sequences, and reference databases is provided in Table S12. Several of these variables were incompletely reported, particularly the numerical interpretation thresholds and the processing of mixed sequences.

### 3.2. Overall Molecular Detection Yield of Valve-Tissue 16S Testing

**Figure 2:**
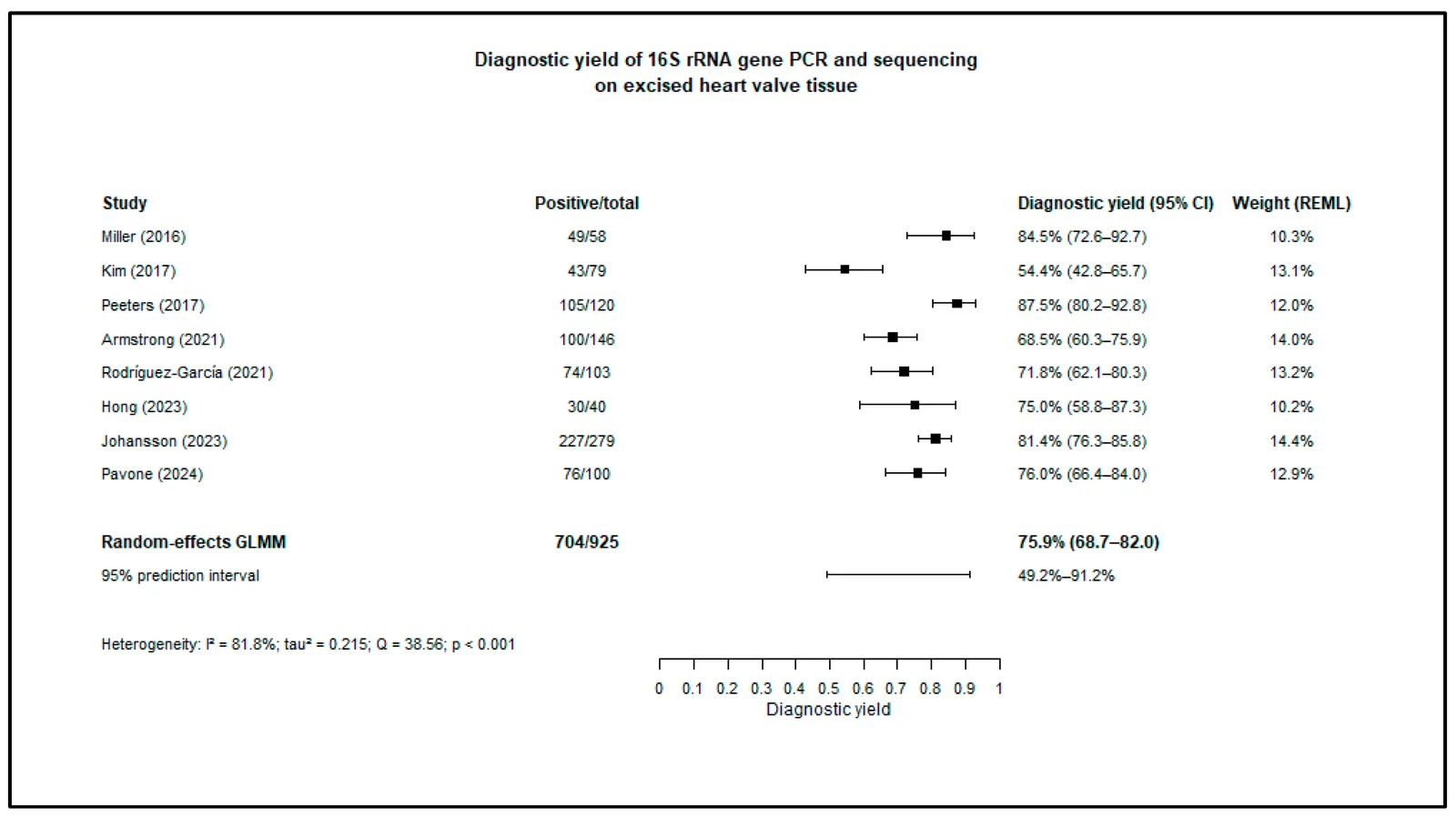
Diagnostic yield of bacterial 16S rRNA gene PCR followed by sequencing on excised heart-valve tissue in infective endocarditis. Squares represent study-specific diagnostic-yield estimates and horizontal lines represent 95% confidence intervals. Study-level confidence intervals were calculated using exact binomial methods. The diamond represents the pooled random-effects estimate obtained using a random-intercept logistic generalized linear mixed model with logit transformation. The 95% prediction interval indicates the range of underlying diagnostic yields that might be expected in a comparable future study. Where study weights are displayed, these are derived from the corresponding inverse-variance random-effects REML model and are provided for visualization; the pooled estimate is the prespecified GLMM result. Abbreviations: CI, confidence interval; GLMM, generalized linear mixed model; PI, prediction interval; REML, restricted maximum likelihood. The studies included in this figure are Miller et al. [4], Kim et al. [5], Peeters et al. [12], Armstrong et al. [6], Rodríguez-García et al. [13], Hong et al. [7], Johansson et al. [8], and Pavone et al. [11].

Across the eight studies included in the primary quantitative synthesis, 704 of 925 patients or episodes had a study-reported molecular-positive result. Study-specific molecular detection yields were 84.5% in Miller et al., 54.4% in Kim et al., 87.5% in Peeters et al., 68.5% in Armstrong et al., 71.8% in Rodríguez-García et al., 75.0% in Hong et al., 81.4% in Johansson et al., and 76.0% in Pavone et al. (Figure 2).

Using a random-intercept logistic generalized linear mixed model, the pooled diagnostic yield of 16S rRNA gene PCR/sequencing performed on excised heart-valve tissue was 75.9% (95% CI, 68.7–82.0%). The corresponding 95% prediction interval was substantially wider, ranging from 49.2% to 91.2%**,** indicating considerable variation in the underlying diagnostic yield that might be expected across comparable clinical settings (Table 2; Figure 2).

Between-study heterogeneity was substantial, with τ^2^ = 0.215, τ = 0.464, and I^2^ = 81.8% (95% CI, 65.4–90.5%). Cochran’s heterogeneity test was significant (Q = 38.56, df = 7, *p* < 0.001). Thus, although the pooled estimate indicated a relatively high overall molecular diagnostic yield, the magnitude of this yield differed substantially between individual cohorts.

An alternative inverse-variance random-effects model using restricted maximum likelihood produced a nearly identical pooled estimate of 75.7% (95% CI, 67.9–82.1%), with a prediction interval of 46.8–91.7%. The close agreement between the GLMM and inverse-variance REML models supported the robustness of the primary estimate to the statistical estimation approach (Table 2).

### 3.3. Robustness, Sensitivity, and Influence Analyses

The primary pooled estimate remained stable in sequential leave-one-out analyses. Exclusion of individual studies resulted in pooled diagnostic yields ranging from 73.7% to 78.3%, indicating that no single study materially determined the magnitude of the overall estimate (Table S2).

The lowest pooled estimate was obtained after exclusion of Peeters et al. [73.7% (95% CI, 66.7–79.8%)], whereas exclusion of Kim et al. yielded the highest estimate [78.3% (95% CI, 72.8–82.9%)]. Excluding Kim et al. also produced the most pronounced reduction in heterogeneity, with I^2^ decreasing from 81.8% in the primary analysis to 69.1% and τ^2^ decreasing from 0.215 to approximately 0.098. This suggested that the comparatively lower yield reported by Kim et al. contributed importantly to between-study heterogeneity, although its removal did not meaningfully change the clinical magnitude of the pooled estimate.

Influence diagnostics were consistent with the leave-one-out findings. Kim et al. had the largest absolute studentized residual (−2.47), DFFITS (−0.89), and Cook’s distance (0.45), and deletion of this study produced the greatest reduction in residual heterogeneity. Peeters et al. also showed comparatively greater influence than most remaining studies, but no observation produced a change sufficient to alter the interpretation of the overall diagnostic yield (Table S3; Figure S1).

Because the Kim publication contained an internal discrepancy between the Results/Figure numerator of 43/79 and the abstract-reported value of 40/79, an additional sensitivity analysis was performed using the latter value. Substitution of 40/79 for 43/79 resulted in a pooled diagnostic yield of 75.6% (95% CI, 67.7–82.1%), compared with 75.9% in the primary analysis. The prediction interval was 46.0–91.9%, and I^2^ was 84.3%. The minimal change in the pooled estimate indicated that the primary findings were insensitive to this within-publication discrepancy.

A further sensitivity analysis excluding the three studies classified as having high overall risk of bias retained five studies comprising 503 participants and 373 molecular-positive results. The pooled diagnostic yield remained essentially unchanged at 75.6% (95% CI, 63.9–84.5%), although heterogeneity remained substantial (I^2^ = 86.9%). The similarity between this estimate and the primary result indicates that the pooled diagnostic yield was not driven by studies judged to be at high overall risk of bias (Table 2).

### 3.4. Risk of Bias

**Figure 3:**
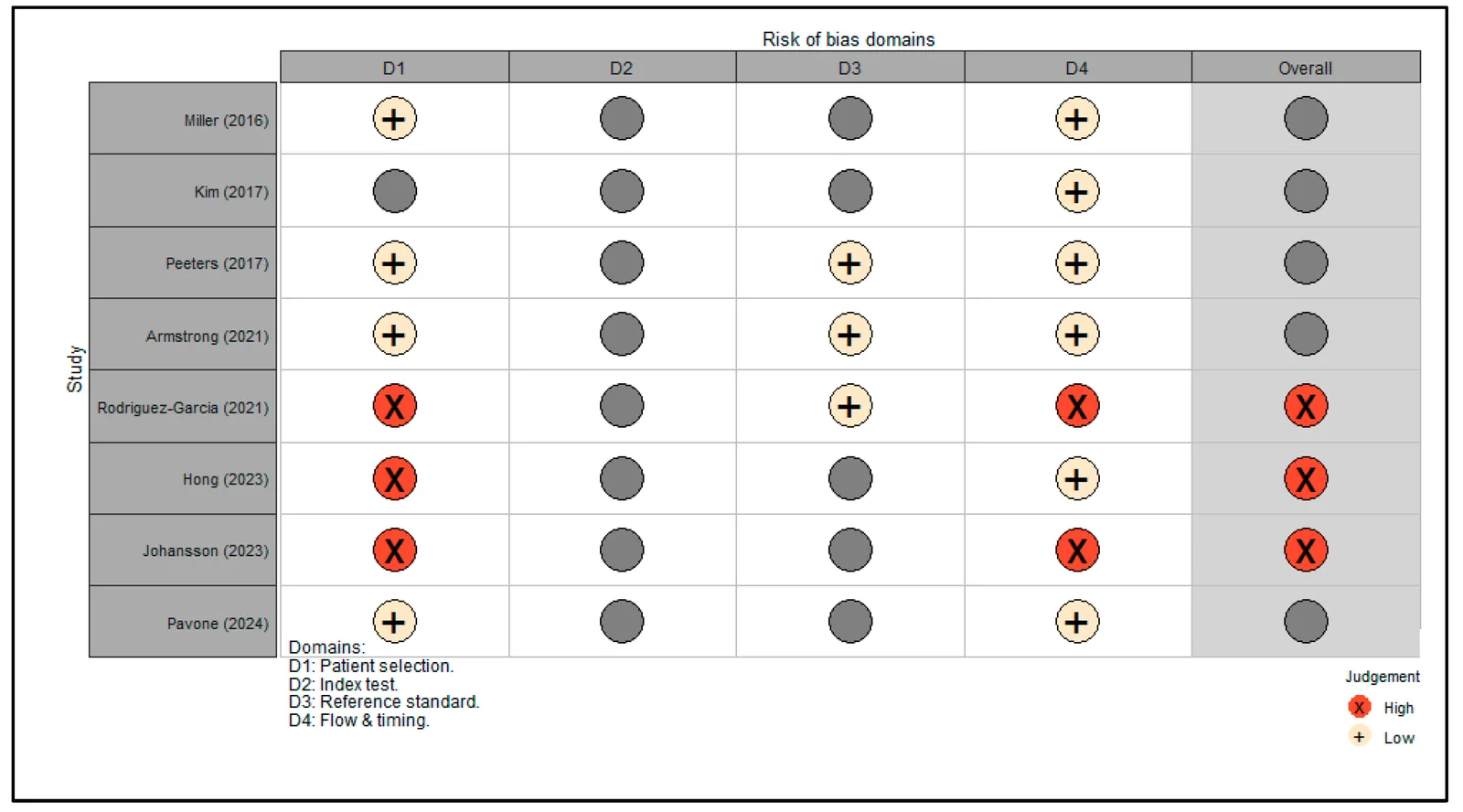
Risk-of-bias assessment of studies included in the primary diagnostic-yield meta-analysis. Risk of bias was evaluated using an adapted QUADAS-2 framework across four domains: patient selection, index test, reference standard, and flow and timing. Each domain was classified as low, unclear, or high risk of bias according to prespecified criteria and information available in the full-text publication. Grey circles without a symbol indicate an unclear risk of bias. Overall risk of bias was classified as high when at least one domain was judged high risk; studies without a high-risk domain but with one or more unclear domains were classified as overall unclear. Detailed domain-level judgments are provided in Table S4. Abbreviation: QUADAS-2, Quality Assessment of Diagnostic Accuracy Studies 2. The studies included in this figure are Miller et al. [4], Kim et al. [5], Peeters et al. [12], Armstrong et al. [6], Rodríguez-García et al. [13], Hong et al. [7], Johansson et al. [8], and Pavone et al. [11].

Among the eight studies included in the primary quantitative synthesis, three were judged to have an overall high risk of bias, while the remaining five were classified as having an unclear overall risk of bias (Figure 3; Table S4).

High overall risk-of-bias judgments were assigned to Rodríguez-García et al., Hong et al., and Johansson et al. Concerns primarily involved patient selection and, in some studies, flow and timing. Patient selection was judged high risk in three studies, while concerns regarding flow and timing were present in two. The index-test domain was judged unclear across all eight studies because methodological reporting did not consistently permit a definitive low-risk judgment. Reference-standard judgments were either low or unclear.

Despite these methodological concerns, exclusion of all studies classified as high risk produced a pooled estimate closely approximating that of the primary analysis, as described above. Consequently, the magnitude of the primary diagnostic-yield estimate was preserved after restriction to studies without an overall high-risk classification.

### 3.5. Diagnostic Yield in Blood-Culture-Negative Infective Endocarditis

Seven studies provided extractable data for a strict blood-culture-negative infective-endocarditis population. These studies included 160 patients or episodes, of whom 81 had a study-reported molecular-positive result.

**Figure 4:**
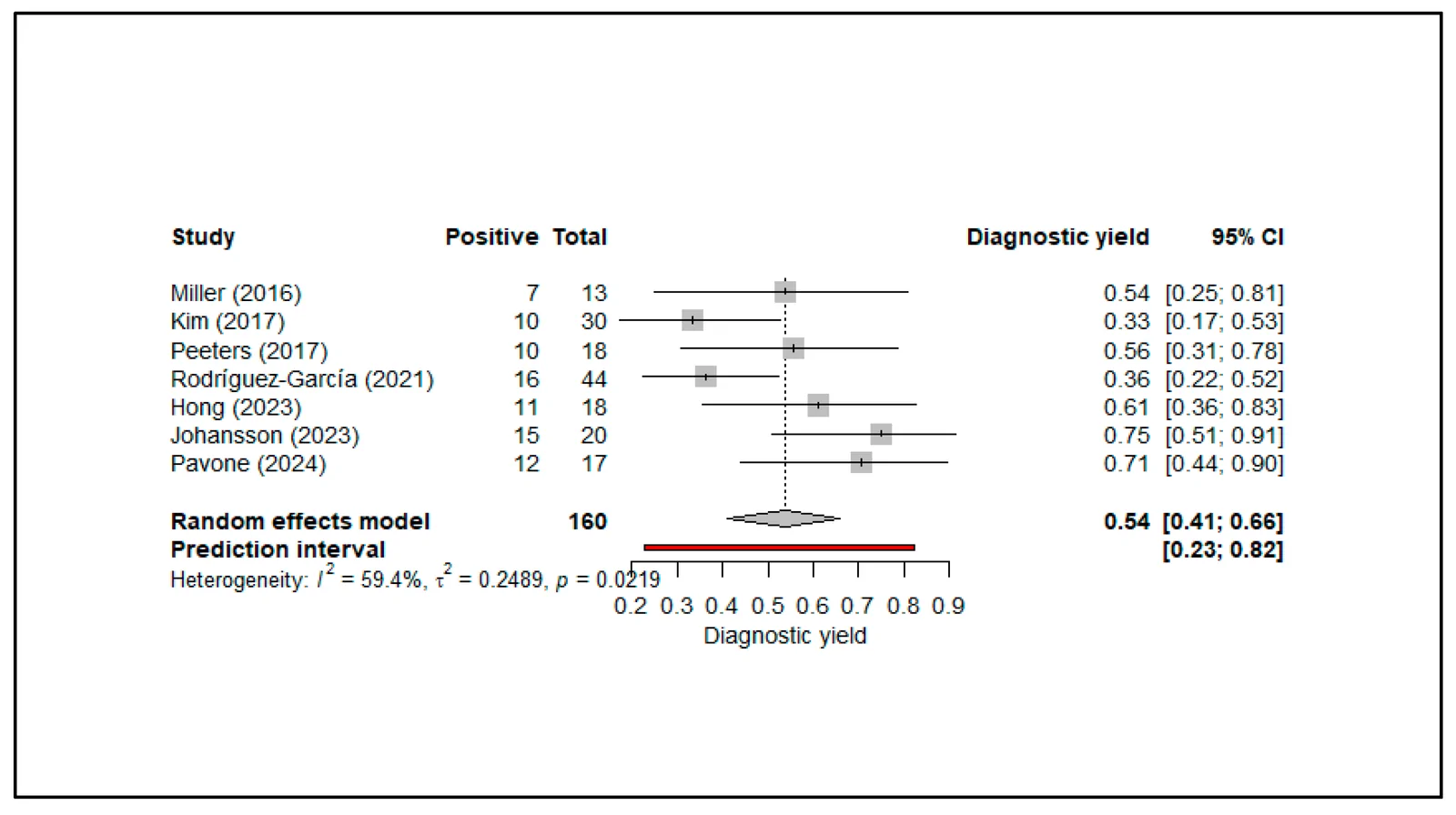
Diagnostic yield of valve-tissue 16S rRNA gene PCR/sequencing in blood-culture-negative infective endocarditis. Diagnostic yield in strict blood-culture-negative infective endocarditis. Strict BCNE was restricted to patients or IE episodes with documented negative blood cultures; cases in which blood-culture status was unavailable were not included in the denominator. Negative results from serology, targeted molecular assays, or valve culture were not required for inclusion; this endpoint does not represent incremental yield after a comprehensive negative diagnostic work-up. The vertical dashed line marks the pooled random-effects estimate; the red rectangle above the x-axis represents the 95% prediction interval. Abbreviations: BCNE, blood-culture-negative infective endocarditis; CI, confidence interval; GLMM, generalized linear mixed model; IE, infective endocarditis; PI, prediction interval. The studies included in this figure are Miller et al. [4], Kim et al. [5], Peeters et al. [12], Rodríguez-García et al. [13], Hong et al. [7], Johansson et al. [8], and Pavone et al. [11].

Study-specific yields varied considerably: 53.8% in Miller et al. (7/13), 33.3% in Kim et al. (10/30), 55.6% in Peeters et al. (10/18), 36.4% in Rodríguez-García et al. (16/44), 61.1% in Hong et al. (11/18), 75.0% in Johansson et al. (15/20), and 70.6% in Pavone et al. (12/17) (Figure 4).

The pooled diagnostic yield in strict BCNE was 53.7% (95% CI, 41.2–65.8%). The 95% prediction interval was 22.7–82.1%, reflecting substantial uncertainty in the molecular yield expected in an individual future BCNE population (Table 2; Figure 4).

Between-study heterogeneity was lower than in the primary overall-yield analysis but remained statistically significant, with τ^2^ = 0.249, I^2^ = 59.4%, and Q = 14.79 (df = 6, *p* = 0.021). Thus, approximately half of patients with blood-culture-negative IE had an etiologically informative molecular result overall, although individual study estimates ranged from approximately one-third to three-quarters of cases.

### 3.6. Strict Incremental Etiologic Yield Beyond Conventional Microbiological Testing

Six studies comprising 834 patients or episodes provided data for the prespecified strict incremental etiologic-yield endpoint, defined as cases in which 16S molecular testing represented the sole positive or sole etiologically informative diagnostic method. A total of 72 strict incremental events were identified.

Study-specific strict incremental yields ranged from 4.7% in Johansson et al. to 15.5% in Rodríguez-García et al. The individual estimates were 10.1% in Kim et al. (8/79), 7.1% in Peeters et al. (9/127), 13.0% in Armstrong et al. (19/146), 15.5% in Rodríguez-García et al. (16/103), 4.7% in Johansson et al. (13/279), and 7.0% in Pavone et al. (7/100).

**Figure 5:**
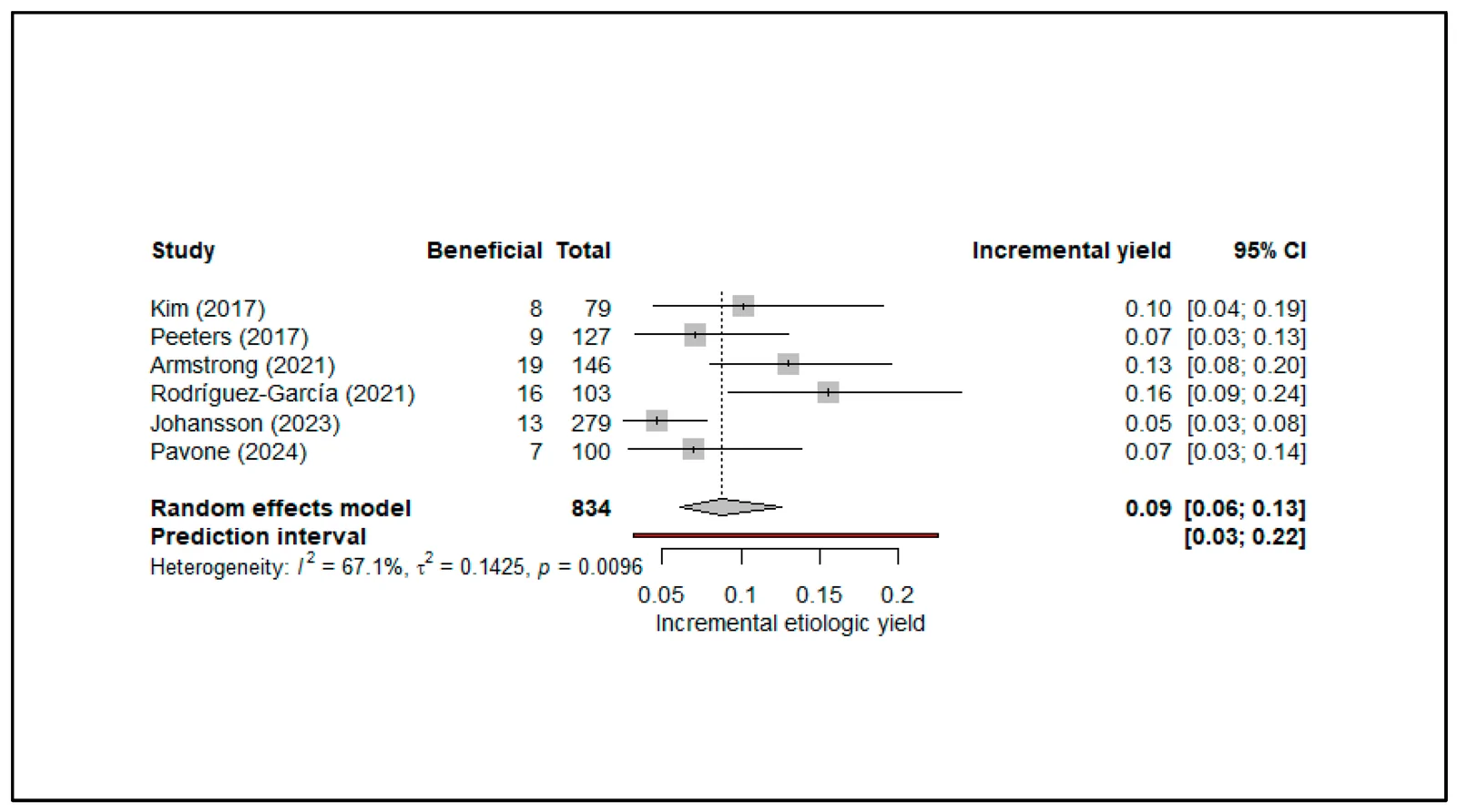
Strict incremental etiologic yield of valve-tissue 16S rRNA gene PCR/sequencing beyond conventional microbiological testing. Strict incremental etiologic yield beyond conventional microbiological testing. Study-specific proportions are shown with exact 95% confidence intervals. Pooled estimates were obtained using random-intercept logistic GLMMs with logit transformation. Diamonds represent pooled estimates, and prediction intervals indicate the expected range of underlying effects in comparable future studies. Strict incremental etiologic yield was defined as molecular testing being the sole positive or sole etiologically informative diagnostic method. Broader study-defined forms of diagnostic benefit were excluded from this endpoint. The vertical dashed line marks the pooled random-effects estimate; the red rectangle above the x-axis represents the 95% prediction interval. Abbreviations: BCNE, blood-culture-negative infective endocarditis; CI, confidence interval; GLMM, generalized linear mixed model; IE, infective endocarditis; PI, prediction interval. The studies included in this figure are Kim et al. [5], Peeters et al. [12], Armstrong et al. [6], Rodríguez-García et al. [13], Johansson et al. [8], and Pavone et al. [11].

The pooled strict incremental etiologic yield was 8.8% (95% CI, 6.1–12.5%), with a 95% prediction interval of 3.1–22.5% (Figure 5; Table 2). Heterogeneity was moderate to substantial (τ^2^ = 0.143; I^2^ = 67.1%), and the heterogeneity test was significant (Q = 15.18, df = 5, *p* = 0.009).

The pooled estimate remained stable across prespecified sensitivity analyses. Exclusion of Peeters et al. yielded an incremental yield of 9.2% (95% CI, 6.1–13.7%), while exclusion of Rodríguez-García et al. resulted in 7.8% (95% CI, 5.4–11.1%). When both studies were excluded simultaneously, the pooled estimate was 8.0% (95% CI, 5.1–12.2%). Prediction intervals remained broad, but the point estimates consistently remained within a narrow range of approximately 8–9% (Table S5).

Organism-specific findings are summarized in Supplementary Table S13. Molecular-only detections included both typical IE pathogens, such as streptococci, staphylococci, and enterococci, and fastidious or intracellular organisms, including *Coxiella burnetii*, *Tropheryma whipplei*, *Bartonella* spp., *Aggregatibacter actinomycetemcomitans*, *Granulicatella adiacens*, and *Abiotrophia defectiva*. Molecular findings were frequently concordant with blood or valve cultures, whereas discordant findings commonly involved skin commensals, possible polymicrobial infections, or organisms considered more etiologically plausible than the corresponding culture isolate. Definitions and reporting of organism-level results differed across studies; therefore, these findings were summarized descriptively and were not pooled.

### 3.7. Diagnostic Accuracy

Only two studies provided sufficient strict bacterial 16S data to reconstruct complete 2 × 2 diagnostic-accuracy tables. Consequently, sensitivity and specificity were summarized descriptively, and no pooled diagnostic-accuracy estimate or hierarchical summary receiver-operating-characteristic model was calculated.

In Miller et al., 46 true-positive, 4 false-positive, 4 false-negative, and 14 true-negative results yielded a sensitivity of 92.0% (95% CI, 80.8–97.8%) and specificity of 77.8% (95% CI, 52.4–93.6%). The positive predictive value was 92.0%, and the negative predictive value was 77.8%.

In Hong et al., 30 true-positive, no false-positive, 10 false-negative, and 11 true-negative results yielded a sensitivity of 75.0% (95% CI, 58.8–87.3%) and specificity of 100.0% (95% CI, 71.5–100.0%). The positive predictive value was 100%, while the negative predictive value was 52.4% (Table S6; Figure S2).

The relatively wide confidence intervals around specificity, particularly in Miller et al., reflected the limited number of reference-negative patients available for evaluation.

### 3.8. Molecular Yield After Negative Blood and Valve Cultures

Four studies provided extractable molecular results in patients for whom both blood cultures and valve cultures were negative. Because of differences in cohort definitions and the precise role attributed to molecular-only results, these data were summarized descriptively and were not quantitatively pooled.

Study-specific molecular yields were 28.6% (95% CI, 13.2–48.7%) in Kim et al. (8/28), 75.0% (95% CI, 42.8–94.5%) in Peeters et al. (9/12), 50.0% (95% CI, 33.4–66.6%) in Armstrong et al. (19/38), and 70.0% (95% CI, 34.8–93.3%) in Pavone et al. (7/10).

Across these studies, 43 molecular-positive cases were observed among 88 patients with both conventional culture modalities negative; however, the aggregate 43/88 value was treated only as a descriptive count and not as a pooled effect estimate (Table S7).

### 3.9. Broader Study-Defined Diagnostic Benefit

Six studies also reported broader measures of diagnostic benefit that extended beyond the prespecified strict incremental etiologic endpoint. These measures were heterogeneous in definition and could include molecular-only etiologic identification, confirmation or clarification of uncertain conventional microbiological results, resolution of discordant findings, or contribution to diagnostic exclusion.

Broad diagnostic-benefit estimates were 10.1% in Kim et al. (8/79), 21.3% in Peeters et al. (27/127), 23.3% in Armstrong et al. (34/146), 31.1% in Rodríguez-García et al. (32/103), 9.0% in Johansson et al. (25/279), and 7.0% in Pavone et al. (7/100). Overall, 133 broad-benefit events were reported among 834 participants.

Because these study-defined endpoints were not conceptually equivalent, the 133/834 aggregate was not meta-analyzed and was retained solely as descriptive evidence (Table S8). This broader endpoint was therefore kept analytically distinct from the strict incremental etiologic yield of 72/834 used in the quantitative synthesis.

Exploratory assessments of funnel-plot asymmetry and small-study effects are presented in Supplementary Figure S3 and Supplementary Table S9; their interpretation is limited by the inclusion of only eight studies.

### 3.10. Subgroup Analysis and Meta-Regression Feasibility

Study-level variables were evaluated for their suitability as potential moderators of the primary diagnostic-yield estimate. However, most clinically or methodologically relevant variables were highly fragmented across the eight studies. Country was unique to each study, study design was distributed across five categories, analysis unit across four categories, and both PCR target and sequencing method were represented by multiple sparsely populated categories.

The only automatically identified binary variable with at least two studies in the smaller group was availability of extractable diagnostic-accuracy data. This variable reflected outcome availability rather than a clinically or methodologically meaningful source of between-study heterogeneity and was therefore not considered an appropriate moderator.

Accordingly, no formal subgroup meta-analysis or meta-regression was undertaken because no meaningful study-level characteristic had sufficient replication to permit reliable estimation of between-group effects (Table S10).

Valve-type-specific molecular outcomes were also reviewed. Miller et al. was the only study providing extractable numerator and denominator data compatible with the primary outcome: molecular testing was positive in 10/17 prosthetic-valve IE cases (58.8%) and, by subtraction from the reported overall result, in 39/41 native-valve IE cases (95.1%) [4]. Peeters et al. reported no statistically significant difference in molecular sensitivity according to valve type, but did not provide extractable stratum-specific numerators and denominators in the main report [12]. The remaining studies reported valve-type distributions without corresponding molecular-positive counts, or used patient-level outcomes in cohorts containing multiple valve specimens. Consequently, separate pooled estimates for native- and prosthetic-valve IE could not be calculated.

Specimen-preservation-specific outcomes were available only from Hong et al. Among patients with active IE, molecular testing was positive in 26/35 fresh-tissue specimens (74.3%) and 4/5 FFPE specimens (80.0%), with no statistically significant difference between specimen types (*p* = 0.78) [7]. The FFPE subgroup was too small to support a reliable comparison, and the remaining studies did not provide separate molecular-positive numerators and denominators for fresh or frozen versus FFPE specimens. Consequently, no pooled specimen-type analysis was performed.

## 4. Discussion

This systematic review and meta-analysis shows that broad-range bacterial 16S rRNA gene PCR followed by sequencing can provide valuable etiological information when performed on surgically excised heart-valve tissue. Across eight studies and 925 patients, surgical cases, or infective-endocarditis episodes, the pooled overall molecular detection yield was 75.9%. However, between-study heterogeneity was substantial (I^2^ = 81.8%), and the 95% prediction interval ranged from 49.2% to 91.2%. Therefore, the pooled estimate should not be interpreted as a universal yield applicable to every clinical setting. Rather, the expected performance of valve-tissue 16S testing may vary considerably according to patient selection, the local diagnostic algorithm, laboratory workflow, and the reference standards used. The molecular detection yield remained important in BCNE, reaching 53.7%, while the strict incremental etiologic yield beyond conventional microbiological testing was 8.8% [4,5,6,7,8,9,10,11,12,13].

Study-specific molecular detection yields varied from 54.4% in Kim et al. to 87.5% in Peeters et al., resulting in substantial statistical heterogeneity and a wide prediction interval [5,12]. This variation is clinically important because it indicates that the diagnostic contribution observed in one center may not be directly transferable to another. Differences in patient selection, prevalence of BCNE, timing of surgery and prior antimicrobial exposure, local diagnostic algorithms, specimen processing, molecular workflows, contamination-control procedures, criteria for interpreting positive results, and reference standards are all plausible contributors to the observed variability [14,15,16].

Nevertheless, the pooled estimate was stable when alternative statistical methods were applied and when individual studies were sequentially excluded. Replacing the Kim et al. numerator of 43/79 with the abstract-reported value of 40/79 also had minimal influence on the overall result.

Available evidence was also insufficient to establish whether molecular yield differs systematically between native- and prosthetic-valve IE. The lower molecular positivity reported for prosthetic-valve cases in the single study with extractable stratified data may reflect differences in specimen availability, sampling, prior treatment, or patient selection and should not be interpreted as a generalizable valve-type effect. Further studies should report molecular results separately for native and prosthetic valves.

Evidence regarding specimen preservation was similarly limited. In the only study reporting separate results, molecular positivity was comparable between fresh and FFPE valve tissue, but only five FFPE specimens were evaluated. Although formalin fixation and paraffin embedding may reduce DNA quality through fragmentation and chemical modification, the available data are insufficient to determine whether FFPE processing materially affects molecular diagnostic yield in IE [17]. Future studies should report results separately for fresh, frozen, and FFPE specimens.

Preoperative antibiotic exposure is an important consideration when interpreting molecular yield, particularly in BCNE. Antibiotics may reduce culture positivity while bacterial DNA remains detectable by 16S rRNA gene PCR [18]. However, molecular detection is not unaffected by treatment duration. In Pavone et al., PCR positivity was 86%, 78%, and 60% after <14, 14–28, and >28 days of preoperative therapy, respectively, compared with valve culture positivity of 76%, 11%, and 20% [11]. Peeters et al. also identified the causative organism by 16S rRNA gene PCR and sequencing in 19/23 patients (82.6%) who had received effective antibiotic therapy for >21 days [12].

These findings support the potential value of molecular testing after antibiotic exposure, but do not establish uniform performance throughout treatment. Exposure timing also matters; antibiotics administered before blood sampling and those administered between blood sampling and surgery may affect comparisons differently [18]. Study-level exposure data are summarized in Supplementary Table S1. A positive molecular result does not establish bacterial viability or ongoing infection and should be interpreted alongside histopathology, operative findings, conventional microbiology, and the clinical course [19]. The BCNE estimate also requires a distinction between blood-culture negativity and unresolved etiology [20,21]. The pooled population was defined by documented negative blood cultures, rather than by a uniformly negative comprehensive diagnostic work-up. Etiological identification by valve culture, serology, or targeted molecular assays did not preclude inclusion in this analysis. Consequently, the pooled yield of 53.7% should not be interpreted as the additional diagnostic yield of 16S rRNA gene PCR and sequencing after exhaustion of other diagnostic methods. Differences in the extent and timing of the diagnostic work-up may also contribute to variation between studies. This endpoint should therefore remain distinct from the separately analyzed strict incremental etiologic yield.

The strict incremental etiologic yield of 8.8% was substantially lower than the overall molecular yield because most molecular-positive results confirmed microorganisms already identified by conventional methods. The strict endpoint included only cases in which 16S testing represented the sole positive or sole etiologically informative method. Although this occurred in fewer than one in ten patients overall, these cases are clinically important because the causative organism might otherwise have remained unidentified. The incremental estimate is also dependent on the scope of the comparator work-up [16,21]. Differences in the use and reporting of serology and organism-specific molecular assays limit comparability between studies. Accordingly, the pooled estimate of 8.8% should be interpreted as incremental identification beyond the documented study-specific microbiological evaluation, not as the expected added yield after a standardized comprehensive diagnostic panel. The microbiological contribution of valve-tissue 16S rRNA gene PCR and sequencing extends beyond the pooled proportion of positive results. Molecular-only findings included organisms that are difficult to recover by routine culture, particularly *Coxiella burnetii*, *Tropheryma whipplei*, and *Bartonella* spp., but also typical cultivable IE pathogens in patients exposed to antibiotics [16,22,23]. Concordance with blood or valve culture can support the etiological relevance of an organism, especially when skin commensals are isolated. Conversely, a discordant molecular result should not automatically override culture findings, because contamination, sampling variation, polymicrobial infection, and persistent nonviable bacterial DNA are alternative explanations [16,19,24]. Organism-specific results must therefore be interpreted together with culture multiplicity, serology or targeted PCR when available, histopathology, antimicrobial exposure, and the clinical presentation. The study-specific findings and interpretive considerations are presented in Supplementary Table S13.

This strict definition should be distinguished from the broader diagnostic-benefit measures reported by individual studies. Armstrong et al., Johansson et al., Peeters et al., and Rodríguez-García et al. included various combinations of molecular-only identification, confirmation of possible contaminants, clarification of discordant culture results, and contribution to diagnostic exclusion [6,8,12,13]. Because these outcomes were not conceptually equivalent, combining them would have overestimated the frequency with which molecular testing independently established the etiology. Separating strict incremental yield from broader study-defined benefit therefore represents an important methodological feature of the present analysis.

Valve-tissue molecular testing also showed potential value when both blood and valve cultures were negative. Across four studies, molecular identification was obtained in 43 of 88 patients, although study-specific yields ranged from 28.6% to 75.0% [5,6,11,12]. These data were not pooled because subgroup definitions and the adjudication of molecular-only findings differed substantially. Nevertheless, they suggest that molecular testing may be particularly useful when both conventional culture methods fail.

The effect of molecular testing on antimicrobial management was less consistently evaluated. Peeters et al. reported treatment changes in 12 of 127 patients, whereas Mularoni et al. described the clinical contribution of a broader prospective workflow incorporating bacterial 16S, fungal 18S, and molecular resistance testing [9,12]. Other studies mainly discussed potential rather than prospectively documented treatment changes [11]. Therefore, diagnostic positivity should not automatically be interpreted as demonstrated therapeutic benefit. Turnaround time, communication with the endocarditis team, availability of susceptibility results, and confidence in the clinical relevance of the detected organism are likely to determine whether treatment is modified [16,25].

Only Miller et al. and Hong et al. provided complete strict bacterial 16S data suitable for diagnostic-accuracy reconstruction [4,7]. Their sensitivities were 92.0% and 75.0%, respectively, while specificities were 77.8% and 100.0%. However, the number of reference-negative patients was small, resulting in wide confidence intervals. The decision not to calculate pooled sensitivity, pooled specificity, or a hierarchical diagnostic-accuracy model was therefore appropriate.

The exploratory studies illustrate the increasing diversity of molecular approaches. Santibáñez et al. used targeted 16S next-generation sequencing, whereas Mularoni et al. combined bacterial 16S and fungal 18S analysis [9,10]. Berinson et al. evaluated a combined 16S/18S assay and a syndromic molecular panel alongside conventional culture [14]. Sinitskaya et al. applied 16S metabarcoding and detected a broad range of bacterial taxa, including organisms that could represent environmental or reagent contamination [15]. These methods may increase analytical sensitivity and taxonomic breadth, but they also complicate the distinction between true pathogens, residual bacterial DNA, mixed microbial signals, and contamination [26,27]. Their specimen-level or mixed-assay outcomes justified retaining them for secondary or exploratory synthesis rather than pooling them with the strict primary analysis.

This review has several strengths. Clinically relevant etiologic identification was separated from technical molecular positivity, and all quantitative numerators and denominators were reverified from the full-text publications. Separate datasets were constructed for overall diagnostic yield, strict BCNE, strict incremental yield, broad diagnostic benefit, both-culture-negative cases, and diagnostic accuracy. The primary estimate was further examined using an alternative REML model, leave-one-out analyses, influence diagnostics, and targeted sensitivity analyses.

Several limitations should also be acknowledged. Only eight studies contributed to the primary meta-analysis, most were single-center observational cohorts, and three were judged to have a high overall risk of bias. The analysis was restricted to surgically managed IE and cannot be generalized to patients without available valve tissue. Differences in reference standards, study populations, molecular methods, and analysis units contributed to substantial heterogeneity. Individual-patient data were unavailable, preventing reliable subgroup analyses according to valve type, antimicrobial exposure, specimen preservation, or sequencing technology. In addition, the search was restricted to Open Access publications from 2016 through 2026. Although these criteria were applied consistently to define a contemporary and directly verifiable evidence base, they may have resulted in the exclusion of otherwise eligible older or non-Open Access studies and may have introduced availability-related selection bias. The pooled estimates should therefore be interpreted as a synthesis of the contemporary Open Access evidence base rather than as an exhaustive synthesis of all historical literature on valve-tissue 16S testing. Although the protocol was formally registered retrospectively, the initial methodological framework was established before the literature search and screening, and subsequent revisions were limited to clarification of the outcome definitions. Because only eight studies contributed to the primary meta-analysis, the assessment of small-study effects was underpowered; these analyses were therefore considered exploratory and are reported in Supplementary Figure S3 and Supplementary Table S9.

The primary pooled estimate represents study-reported molecular detection rather than uniformly adjudicated patient-level etiological relevance. Criteria for classifying contaminants, unresolved or genus-level sequences, discordant findings, and persistent bacterial DNA were inconsistently reported across studies, precluding standardized retrospective reclassification. We therefore retained the original study classifications, excluded explicitly reported contaminants when possible, and present the study-specific handling of potentially ambiguous findings in Table S11.

Reporting of molecular laboratory workflows was incomplete and inconsistent. The taxonomic resolution of 16S rRNA gene sequencing depends on the targeted region, amplicon length, sequence quality, reference database, and similarity threshold used for identification. Short or partial sequences may not reliably distinguish closely related species, particularly within groups such as viridans-group streptococci and coagulase-negative staphylococci [28,29]. Sanger sequencing also has limited ability to resolve polymicrobial specimens because overlapping chromatograms may preferentially identify the predominant organism or produce mixed, genus-level, or uninterpretable results [30]. Only some studies reported mixed-sequence deconvolution or supplementary next-generation sequencing. Because several studies did not provide the exact targeted region, primer sequences, numerical similarity thresholds, database, or procedures used for mixed sequences, species-level identifications could not be independently standardized or re-adjudicated. These methodological limitations, summarized in Table S12, contributed to the unclear risk-of-bias judgments for the index-test domain and may partly explain the variability in molecular detection yield.

Preoperative antibiotic exposure was also reported inconsistently, particularly regarding treatment duration and timing relative to blood sampling and surgery. Consequently, its contribution to between-study heterogeneity and the pooled molecular detection yield could not be reliably quantified.

## 5. Conclusions

Valve-tissue 16S rRNA gene PCR and sequencing may be a useful complementary diagnostic method in selected patients undergoing surgery for infective endocarditis, particularly when conventional microbiological findings are negative or inconclusive. The pooled overall molecular detection yield was 75.9%, while the yield among patients with documented negative blood cultures was 53.7% and the strict incremental etiologic yield beyond the documented study-specific microbiological work-up was 8.8%. However, substantial between-study heterogeneity and wide prediction intervals indicate that the expected diagnostic yield may vary markedly across clinical settings, and heterogeneity remained considerable in sensitivity analyses. The magnitude and interpretation of molecular yield depend on patient selection, preoperative antimicrobial exposure, laboratory methodology, and the diagnostic algorithm used at each center. Molecular findings should therefore be integrated with cultures, histopathology, serology, targeted molecular assays, and the clinical context. Standardized prospective studies are needed to define the patients most likely to benefit and to determine whether additional etiological identification improves antimicrobial management and clinical outcomes.

## Figures and Tables

**Table 1:** Characteristics of studies included in the primary quantitative synthesis of 16S rRNA gene PCR/sequencing on excised heart-valve tissue.

Study	Country	Study Design	Analysis Unit	Primary Population	Molecular Method	Positive/Total	Diagnostic Yield
**Miller et al. (2016)** [4]	Canada	Prospective diagnostic cohort	Patient	Suspected native/prosthetic IE; definite + possible IE used for yield	Fast broad-range 16S rRNA gene PCR; Sanger sequencing	49/58	84.5%
**Kim et al. (2017)** [5]	South Korea	Prospective cohort	Patient/episode	Surgical IE; 65 definite and 14 possible episodes	Direct broad-range 16S rRNA gene PCR; Sanger sequencing	43/79	54.4%
**Peeters et al. (2017)** [12]	Belgium	Prospective cohort	Patient	Surgical suspected IE; postoperative definite-IE subgroup	Broad-range 16S rRNA gene PCR; amplicon sequencing	105/120	87.5%
**Armstrong et al. (2021)** [6]	Germany	Retrospective cohort	Patient/case	Histopathologically/macroscopically confirmed surgical IE	Broad-range 16S rRNA gene PCR; sequence-based identification	100/146	68.5%
**Rodríguez-García et al. (2021)** [13]	Spain	Retrospective cohort	Patient/episode	Selected surgical IE subset undergoing 16S PCR	Broad-range 16S rRNA gene PCR; Sanger sequencing	74/103	71.8%
**Hong et al. (2023)** [7]	United States	Retrospective diagnostic cohort	Patient	Patients undergoing valve testing; active IE subgroup	Broad-range 16S rRNA gene PCR/sequencing	30/40	75.0%
**Johansson et al. (2023)** [8]	Sweden	Retrospective cohort	IE episode	Adult IE episodes undergoing valve culture and 16S testing	Broad-range 16S rRNA gene PCR; Sanger sequencing	227/279	81.4%
**Pavone et al. (2024)** [11]	Italy	Prospective observational cohort	Patient/episode	Surgical definite or suspected native/prosthetic IE	Broad-range bacterial 16S rRNA gene PCR; sequence-based identification	76/100	76.0%

Abbreviations: 16S PCR followed by sequence-based identification. Where the total published cohort differed from the denominator used for the primary meta-analysis, the meta-analytic denominator is shown. More detailed population definitions, reference standards, specimen characteristics, and molecular workflows are provided in Table S1.

**Table 2:** Summary of the primary meta-analysis, secondary quantitative syntheses, and key sensitivity analyses.

Analysis	Studies	N	Events	Pooled Yield, % (95% CI)	95% Prediction Interval	τ^2^	I^2^
**Primary GLMM**	8	925	704	75.9 (68.7–82.0)	49.2–91.2	0.215	81.8%
**Inverse-variance REML**	8	925	704	75.7 (67.9–82.1)	46.8–91.7	0.248	81.8%
**Kim abstract value, 40/79**	8	925	701	75.6 (67.7–82.1)	46.0–91.9	0.258	84.3%
**Excluding high-risk studies**	5	503	373	75.6 (63.9–84.5)	33.6–95.0	0.345	86.9%
**Strict BCNE**	7	160	81	53.7 (41.2–65.8)	22.7–82.1	0.249	59.4%
**Strict incremental etiologic yield**	6	834	72	8.8 (6.1–12.5)	3.1–22.5	0.143	67.1%

Abbreviations: τ^2^ estimated between-study variance; I^2^, proportion of total observed variability attributable to between-study heterogeneity rather than sampling error. Notes: Pooled proportions were estimated using a random-intercept logistic GLMM with logit transformation unless otherwise specified. The inverse-variance analysis used a random-effects model with REML estimation of between-study variance. The 95% PI represents the range of underlying effects expected in a comparable future study and is therefore wider than the 95% CI around the pooled mean. The Kim sensitivity analysis replaced the primary numerator of 43/79, reported in the article Results/Figure, with the abstract-reported value of 40/79. The risk-of-bias sensitivity analysis excluded studies classified as having high overall risk of bias. Strict BCNE included only patients or episodes with documented negative blood cultures. Strict incremental etiologic yield was defined as cases in which molecular testing constituted the sole positive or sole etiologically informative diagnostic method.

## Data Availability

The datasets generated during the current study are available from the corresponding author on reasonable request.

## Supplementary Materials

The following supporting information can be downloaded at: https://www.mdpi.com/article/10.3390/diagnostics16193125/s1, File S1 contains Table S1: Detailed methodological and clinical characteristics of studies included in the systematic review; Table S2: Leave-one-out analysis of the primary diagnostic-yield meta-analysis; Table S3: Influence diagnostics for the primary diagnostic-yield meta-analysis; Table S4: Domain-level risk-of-bias judgments for studies included in the primary meta-analysis; Table S5: Sensitivity analyses of the strict incremental etiologic-yield meta-analysis; Table S6: Diagnostic-accuracy estimates for valve-tissue 16S rRNA gene PCR/sequencing; Table S7: Molecular diagnostic yield among patients with both negative blood cultures and negative valve cultures; Table S8: Study-defined broad diagnostic benefit of valve-tissue 16S molecular testing; Table S9: Exploratory tests for funnel-plot asymmetry and small-study effects; Table S10: Feasibility assessment of candidate study-level moderators for subgroup analysis or meta-regression; Table S11: Study-level definition of molecular positivity, handling of potentially ambiguous results, and documented non-index microbiological work-up in the primary meta-analysis; Table S12: Detailed laboratory characteristics of valve-tissue 16S PCR and sequencing methods used in studies included in the primary meta-analysis; Table S13: Study-reported organism-specific findings from valve-tissue 16S rRNA gene PCR and sequencing in studies included in the primary meta-analysis; Figure S1: Influence diagnostics for individual studies included in the primary diagnostic-yield meta-analysis. Panels display externally studentized residuals (rstudent), DFFITS, Cook’s distance (cook.d), covariance ratios (cov.r), estimated between-study variance after study deletion (tau2.del), residual heterogeneity after study deletion (QE.del), leverage (hat), and model weight. Studies are numbered according to their order in the primary meta-analysis: 1, Miller; 2, Kim; 3, Peeters; 4, Armstrong; 5, Rodríguez-García; 6, Hong; 7, Johansson; and 8, Pavone. Influence diagnostics were obtained from a random-effects model fitted to logit-transformed proportions using REML estimation. Metrics were interpreted jointly; no study was automatically excluded on the basis of a single diagnostic measure. Abbreviations: DFFITS, standardized change in fitted value following study deletion; QE, residual heterogeneity statistic; REML, restricted maximum likelihood; Figure S2: Sensitivity and specificity of valve-tissue bacterial 16S rRNA/rDNA PCR/sequencing in studies with extractable diagnostic-accuracy data. Point estimates and exact binomial 95% confidence intervals are shown separately for sensitivity and specificity. Only studies providing reconstructable complete 2 × 2 tables for strict bacterial 16S testing were included. No pooled estimate or hierarchical summary receiver-operating-characteristic model was calculated because only two eligible studies were available. Abbreviation: CI, confidence interval; Figure S3: Funnel plot for the primary diagnostic-yield meta-analysis. The plot provides an exploratory graphical assessment of potential small-study effects among the eight studies included in the primary quantitative synthesis. Formal assessments using Egger’s regression, Begg-Mazumdar rank correlation, and Peters’ regression are reported in Table S9. Because fewer than 10 studies were available, funnel-plot interpretation and formal asymmetry tests were considered exploratory and underpowered; apparent symmetry or non-significant tests should not be interpreted as evidence excluding publication bias. File S2: PRISMA 2020 checklist.

## Abbreviations

The following abbreviations are used in this manuscript:

BCNE	Blood-culture-negative infective endocarditis
CI	Confidence interval
DNA	Deoxyribonucleic acid
FN	False negative
FP	False positive
GLMM	Generalized linear mixed model
HSROC	Hierarchical summary receiver-operating-characteristic
IE	Infective endocarditis
I^2^	Proportion of total variability attributable to between-study heterogeneity
NGS	Next-generation sequencing
NPV	Negative predictive value
PCR	Polymerase chain reaction
PI	Prediction interval
PPV	Positive predictive value
PRISMA	Preferred Reporting Items for Systematic Reviews and Meta-Analyses
QUADAS-2	Quality Assessment of Diagnostic Accuracy Studies 2
REML	Restricted maximum likelihood
rDNA	Ribosomal deoxyribonucleic acid
rRNA	Ribosomal ribonucleic acid
TN	True negative
TP	True positive
τ	Estimated between-study standard deviation
τ^2^	Estimated between-study variance
