# Supplementary material for: Diagnostic Yield and Incremental Value of 16S rRNA Gene PCR and Sequencing on Excised Heart-Valve Tissue in Infective Endocarditis: A Systematic Review and Meta-Analysis
Source: Diagnostics (Basel). 2026 Sep 25;16(19):3125. doi: 10.3390/diagnostics16193125 (PMC13664324; doi:10.3390/diagnostics16193125)
Supplement: Supplementary file 1 [file diagnostics-16-03125-s001.zip › Supplementary File S1.pdf]

Table S1. Detailed methodological and clinical characteristics of studies included in the systematic review.

Study (country)	Design / period	Population / IE standard	N / valve specimens	Specimen	16S assay / sequencing	Pre-op antibiotics	Role in review
**Sinitskaya et al., 2026 (Russia)**	Prospective observational; period NR	Surgical IE; Duke criteria	16 pts / 20 valves	Native valves	Broad-range 16S; Illumina MiSeq	12/16 (75%)	Exploratory targeted-NGS
**Berinson et al., 2025 (Germany)**	Prospective diagnostic; 2023–2024	Explanted valves; composite clinical/microbiological standard	97 pts / 100 valves	70 native; 30 prosthetic	16S/18S SepsiTest; Sanger	NR	Secondary mixed-assay
**Pavone et al., 2024 (Italy)**	Prospective cohort; 2020–2023	Definite/suspected surgical IE; guideline-based diagnosis	100 pts / 100 valves	Native/prosthetic valves	Broad-range bacterial 16S; sequencing	Median preoperative treatment duration: 13 days (IQR, 7–33). Duration categories were reported for 98/100 patients; duration was uncertain in 2/100.	Primary meta-analysis
**Hong et al., 2023 (United States)**	Retrospective diagnostic; 2020–2022	Active/cured IE and non-IE controls; clinicopathological standard	54 pts / 54 valves	Active-IE subset: 35 fresh valve-tissue specimens and 5 FFPE valve-tissue specimens	16S V1–V3; Sanger ± NGS	33/40 (82.5%) received antibiotics within 4 weeks before surgery. Treatment duration was reported as 18 days (IQR, 6–38), with the central	Primary meta-analysis

						statistic labelled "mean" in the original report.	
**Johansson et al., 2023 (Sweden)**	Retrospective cohort; 2012–2021	Adult surgical IE; ESC/Duke adjudication	279 IE episodes / 279 valves	Excised valves	Broad-range 16S rDNA; Sanger	The proportion exposed and preoperative treatment duration were not quantified in the main report. Patients undergoing surgery after completion of antibiotic treatment were excluded.	Primary meta-analysis
**Mularoni et al., 2023 (Italy)**	Prospective diagnostic; 2012–2021	Definite surgical IE + non-IE controls	189 pts / 189 valves	Excised valves	Bacterial 16S + fungal 18S; Sanger	NR	Secondary mixed-assay
**Santibáñez et al., 2022 (Spain)**	Observational molecular cohort; period NR	Surgical IE; clinical diagnosis	27 pts / 22 valves †	18 native; 4 prosthetic	16S V3–V4; Illumina MiSeq	NR	Exploratory targeted-NGS
**Armstrong et al., 2021 (Germany)**	Retrospective cohort; 2015–2018	Surgical IE; histopathology/operative confirmation	151 pts / 182 valves	159 native; 23 prosthetic	Broad-range 16S rDNA; sequencing	Most patients received prolonged antibiotic treatment before surgery; the proportion exposed and	Primary meta-analysis

						treatment duration were not quantified in the main report.	
**Rodríguez-García et al., 2021 (Spain)**	Retrospective cohort; 2015–2019	Clinical IE; surgical subset	266 IE pts / 154 valves ‡	Heart-valve tissue	Broad-range 16S rDNA; Sanger	All surgical patients received antibiotics before surgery; mean duration: 10.3 ± 12.8 days (range, 1–105), reported for the surgical cohort rather than separately for the PCR-tested subgroup.	Primary meta-analysis
**Kim et al., 2017 (South Korea)**	Prospective cohort; 2013–2015	Surgical definite/possible IE; modified Duke	80 pts / 80 valves	Heart-valve tissue	Broad-range 16S rDNA; Sanger	79/79 (100%); median preoperative treatment duration: 5 days (range, 1–150).	Primary meta-analysis
**Peeters et al., 2017 (Belgium)**	Prospective cohort; 2013–2016	Suspected surgical IE; postoperative definite IE standard	127 pts / 127 valves	Resected valves	Broad-range 16S; amplicon sequencing	103/120 (85.8%) received effective preoperative antibiotic therapy, defined as treatment and dosing conforming	Primary meta-analysis

						to international guidelines. Twenty-three patients received effective therapy for >21 days; molecular identification remained positive in 19/23 (82.6%).	
**Miller et al., 2016 (Canada)**	Prospective diagnostic; 2009–2014	Suspected native/prosthetic IE; modified Duke + adjudication	68 pts / 68 valves	Heart-valve tissue	16S V1–V3; Sanger	All definite/possible IE cases received appropriate perioperative antibiotics. Preoperative treatment duration was not separately summarized.	Primary meta-analysis

Abbreviations: ESC, European Society of Cardiology; FFPE, formalin-fixed paraffin-embedded; IE, infective endocarditis; NGS, next-generation sequencing; NR, not reported; PCR, polymerase chain reaction; rDNA, ribosomal DNA; rRNA, ribosomal RNA.
Notes: Sample sizes refer to the relevant published cohort and, where applicable, the specific patient-, episode-, case-, or specimen-level denominator contributing to the review. Consequently, sample sizes in this table should not be summed to derive the denominator of the primary meta-analysis. The primary quantitative synthesis included studies providing sufficiently comparable patient- or episode-level bacterial 16S PCR/sequencing data. Studies using targeted NGS/metabarcoding, predominantly specimen-level outcomes, or combined bacterial 16S/fungal 18S assays were retained for secondary or exploratory synthesis but were not pooled with the strict primary bacterial 16S analysis. "Role in review" indicates whether each study contributed to the primary meta-analysis or only to a secondary/exploratory component.

Table S2. Leave-one-out analysis of the primary diagnostic-yield meta-analysis.

Study excluded	Studies remaining	Pooled yield, % (95% CI)	τ^2	I^2
**Peeters (2017)**	7	73.7 (66.7–79.8)	0.150	78.9%
**Miller (2016)**	7	74.7 (66.8–81.3)	0.212	83.2%
**Johansson (2023)**	7	74.9 (66.5–81.8)	0.235	80.5%
**Pavone (2024)**	7	76.0 (67.5–82.8)	0.256	84.4%
**Hong (2023)**	7	76.0 (67.9–82.7)	0.247	84.4%
**Rodríguez-García (2021)**	7	76.5 (68.3–83.2)	0.251	84.2%
**Armstrong (2021)**	7	77.0 (69.1–83.5)	0.234	82.5%
**Kim (2017)**	7	78.3 (72.8–82.9)	0.098	69.1%

Abbreviations: CI, confidence interval; GLMM, generalized linear mixed model; *k*, number of studies remaining after exclusion; τ^2, between-study variance; I^2, percentage of total variability attributable to between-study heterogeneity.
Notes: Each row represents refitting of the primary random-effects diagnostic-yield model after sequential exclusion of one study. Pooled proportions were estimated using the same random-intercept logistic GLMM specification as in the primary analysis. Leave-one-out analyses were used to assess whether the pooled estimate or heterogeneity was disproportionately dependent on any single study. Changes in the pooled estimate should therefore be interpreted relative to the primary pooled yield of 75.9%.

Table S3. Influence diagnostics for the primary diagnostic-yield meta-analysis.

Study	Studentized residual	DFFITS	Cook's distance	τ^2 after deletion	Q after deletion	REML weight
**Miller (2016)**	0.954	0.323	0.105	0.250	35.78	10.3%
**Kim (2017)**	−2.474	−0.893	0.451	0.118	19.39	13.1%
**Peeters (2017)**	1.714	0.652	0.337	0.180	28.44	12.0%
**Armstrong (2021)**	−0.702	−0.288	0.091	0.276	34.34	14.0%
**Rodríguez-García (2021)**	−0.370	−0.155	0.028	0.294	37.88	13.2%
**Hong (2023)**	−0.065	−0.033	0.001	0.291	38.56	10.2%
**Johansson (2023)**	0.665	0.262	0.075	0.274	30.73	14.4%
**Pavone (2024)**	0.025	−0.003	<0.001	0.300	38.51	12.9%

Abbreviations: Cook's D, Cook's distance; DFBETAS, standardized change in the fitted model coefficient after deletion of an individual study; DFFITS, standardized change in the fitted value following study deletion; QE.del, residual heterogeneity statistic after deletion of the study; rstudent, externally studentized residual; τ^2.del, estimated between-study variance after deletion of the study.
Notes: Influence diagnostics were calculated using a random-effects model fitted to logit-transformed proportions with REML estimation. The hat value represents study leverage, whereas the reported weight reflects the study's contribution to the fitted inverse-variance model. Larger absolute studentized residuals and DFFITS values, larger Cook's distances, or marked changes in τ^2 after study deletion may indicate greater influence. These diagnostics were evaluated collectively rather than using any single metric as an automatic exclusion criterion. Studies identified as relatively influential were retained in the primary analysis unless otherwise specified in sensitivity analyses.

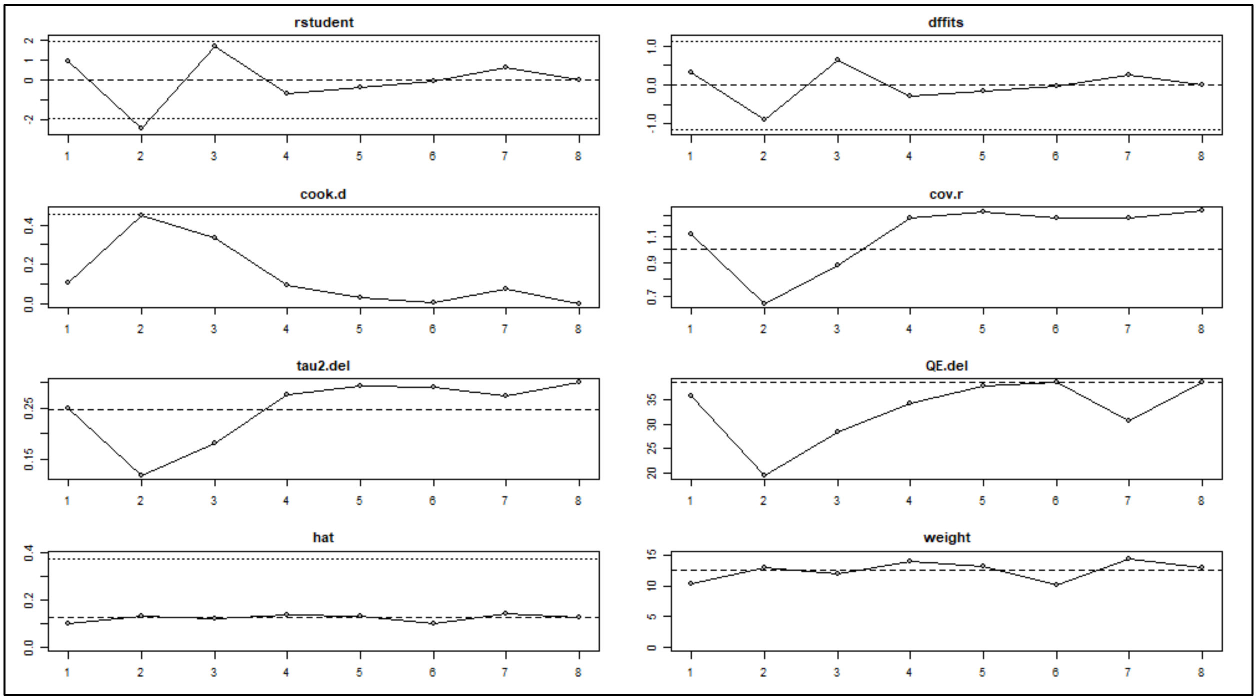

Figure S1. Influence diagnostics for individual studies included in the primary diagnostic-yield meta-analysis. Panels display externally studentized residuals (rstudent), DFFITS, Cook's distance (cook.d), covariance ratios (cov.r), estimated between-study variance after study deletion (tau2.del), residual heterogeneity after study deletion (QE.del), leverage (hat), and model weight. Studies are numbered according to their order in the primary meta-analysis: 1, Miller; 2, Kim; 3, Peeters; 4, Armstrong; 5, Rodríguez-García; 6, Hong; 7, Johansson; and 8, Pavone. Influence diagnostics were obtained from a random-effects model fitted to logit-transformed proportions using REML estimation. Metrics were interpreted jointly; no study was automatically excluded on the basis of a single diagnostic measure. **Abbreviations:** DFFITS, standardized change in fitted value following study deletion; QE, residual heterogeneity statistic; REML, restricted maximum likelihood.

Table S4. Domain-level risk-of-bias judgments for studies included in the primary meta-analysis.

Study	Patient selection	Index test	Reference standard	Flow and timing	Overall
**Miller (2016)**	Low	Unclear	Unclear	Low	Unclear
**Kim (2017)**	Unclear	Unclear	Unclear	Low	Unclear
**Peeters (2017)**	Low	Unclear	Low	Low	Unclear
**Armstrong (2021)**	Low	Unclear	Low	Low	Unclear
**Rodríguez-García (2021)**	High	Unclear	Low	High	High
**Hong (2023)**	High	Unclear	Unclear	Low	High
**Johansson (2023)**	High	Unclear	Unclear	High	High
**Pavone (2024)**	Low	Unclear	Unclear	Low	Unclear

Abbreviations: D1, patient selection; D2, index test; D3, reference standard; D4, flow and timing; QUADAS-2, Quality Assessment of Diagnostic Accuracy Studies 2.
Notes: Risk of bias was assessed using an adapted QUADAS-2 framework. Each domain was rated as low, unclear, or high risk of bias based on the information reported in the full-text article. An overall high-risk judgment was assigned

when at least one domain was rated high risk. Studies with no high-risk domain but at least one unclear domain were classified as overall unclear. “Unclear” indicates insufficient reporting to support either a low- or high-risk judgment and should not be interpreted as equivalent to high risk. The index-test domain remained rated as unclear when one or more laboratory variables required for complete assessment—particularly sequence-quality criteria, numerical similarity thresholds, or the handling of mixed sequences—were insufficiently reported. The additional descriptive information presented in Table S12 improves methodological transparency but does not replace information that was unavailable in the original study reports.

Table S5. Sensitivity analyses of the strict incremental etiologic-yield meta-analysis.

Analysis	Studies	N	Events	Pooled yield, % (95% CI)	Prediction interval	τ^2	I^2
**Primary strict incremental model**	6	834	72	8.8 (6.1–12.5)	3.1–22.5	0.143	67.1%
**Excluding Peeters**	5	707	63	9.2 (6.1–13.7)	2.7–27.2	0.168	71.9%
**Excluding Rodríguez-García**	5	731	56	7.8 (5.4–11.1)	2.9–19.0	0.096	58.7%
**Excluding both**	4	604	47	8.0 (5.1–12.2)	2.2–25.4	0.127	68.2%

Abbreviations: CI, confidence interval; GLMM, generalized linear mixed model; PI, prediction interval; *k*, number of studies; *N*, total number of patients or IE episodes; τ^2, between-study variance; I^2, proportion of total variability attributable to between-study heterogeneity.
Notes: Strict incremental etiologic yield was defined as the proportion of patients or IE episodes in whom bacterial 16S molecular testing constituted the sole positive or sole etiologically informative diagnostic method. Sensitivity models excluded Peeters et al., Rodríguez-García et al., or both studies to assess the impact of study-specific denominator or selection considerations. All models used the same random-intercept logistic GLMM framework as the primary incremental-yield analysis.

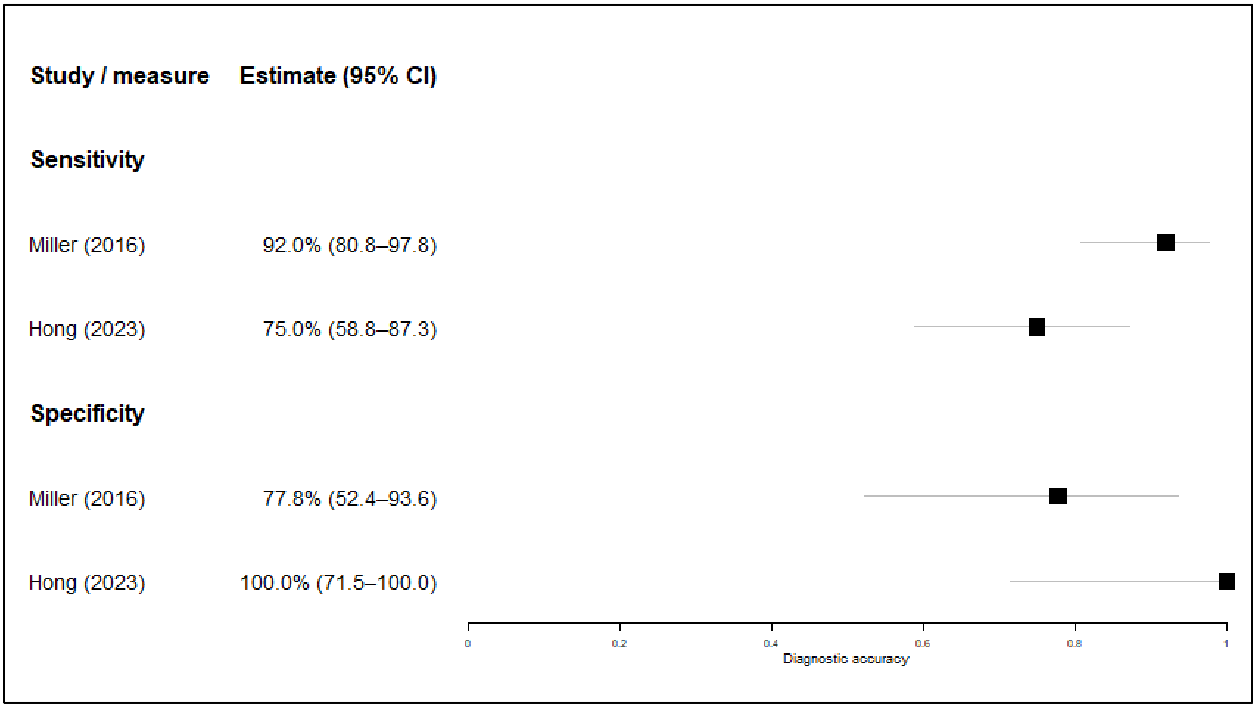

Figure S2. Sensitivity and specificity of valve-tissue bacterial 16S rRNA/rDNA PCR/sequencing in studies with extractable diagnostic-accuracy data. Point estimates and exact binomial 95% confidence intervals are shown separately for sensitivity and specificity. Only studies providing reconstructable complete 2×2 tables for strict bacterial 16S testing were included. No pooled estimate or hierarchical summary receiver-operating-characteristic model was calculated because only two eligible studies were available. **Abbreviation:** CI, confidence interval.

Table S6. Diagnostic-accuracy estimates for valve-tissue 16S rRNA gene PCR/sequencing.

Study	TP	FP	FN	TN	Sensitivity, % (95% CI)	Specificity, % (95% CI)	PPV	NPV
**Miller (2016)**	46	4	4	14	92.0 (80.8–97.8)	77.8 (52.4–93.6)	92.0%	77.8%
**Hong (2023)**	30	0	10	11	75.0 (58.8–87.3)	100.0 (71.5–100.0)	100.0%	52.4%

Abbreviations: CI, confidence interval; FN, false negative; FP, false positive; NPV, negative predictive value; PPV, positive predictive value; TN, true negative; TP, true positive.
Notes: Sensitivity was calculated as TP/(TP+FN), specificity as TN/(TN+FP), PPV as TP/(TP+FP), and NPV as TN/(TN+FN). Confidence intervals for sensitivity and specificity were calculated using exact binomial methods. Only studies permitting reconstruction of a complete 2×2 table for strict bacterial 16S testing were included. Studies reporting combined bacterial 16S/fungal 18S results were not included in the strict accuracy analysis. Because only two eligible strict bacterial 16S studies were available, sensitivity and specificity were not quantitatively pooled and no bivariate or hierarchical summary receiver-operating-characteristic model was fitted.

Table S7. Molecular diagnostic yield among patients with both negative blood cultures and negative valve cultures.

Study	Positive / total	Diagnostic yield, % (95% CI)

**Kim (2017)**	8/28	28.6 (13.2–48.7)
**Peeters (2017)**	9/12	75.0 (42.8–94.5)
**Armstrong (2021)**	19/38	50.0 (33.4–66.6)
**Pavone (2024)**	7/10	70.0 (34.8–93.3)

Abbreviations: CI, confidence interval; IE, infective endocarditis.
Notes: This analysis was restricted to study-defined patients or episodes in whom both blood cultures and valve cultures were negative. Molecular yield represents the proportion with a clinically relevant valve-tissue molecular result. Exact binomial 95% confidence intervals are shown. Because definitions and ascertainment of this subgroup varied across studies, results were summarized descriptively and no pooled meta-analytic estimate was calculated. The aggregate number of molecular-positive cases across studies is presented only as a descriptive count and should not be interpreted as a pooled proportion.

Table S8. Study-defined broad diagnostic benefit of valve-tissue 16S molecular testing.

Study	Cases / total	Broad diagnostic benefit, % (95% CI)
**Kim (2017)**	8/79	10.1 (4.5–19.0)
**Peeters (2017)**	27/127	21.3 (14.5–29.4)
**Armstrong (2021)**	34/146	23.3 (16.7–31.0)
**Rodríguez-García (2021)**	32/103	31.1 (22.3–40.9)
**Johansson (2023)**	25/279	9.0 (5.9–12.9)
**Pavone (2024)**	7/100	7.0 (2.9–13.9)

Abbreviations: CI, confidence interval; IE, infective endocarditis.
Notes: Broad diagnostic benefit reflects the definition reported by each individual study and may include molecular-only etiologic identification, confirmation or clarification of uncertain conventional microbiological findings, resolution of discordant results, or contribution to diagnostic exclusion. These definitions were not considered clinically or methodologically equivalent across studies. Therefore, study-specific proportions are presented descriptively and were not quantitatively pooled. Broad diagnostic benefit is distinct from the prespecified strict incremental etiologic-yield endpoint, which required molecular testing to represent the sole positive or sole etiologically informative diagnostic method.

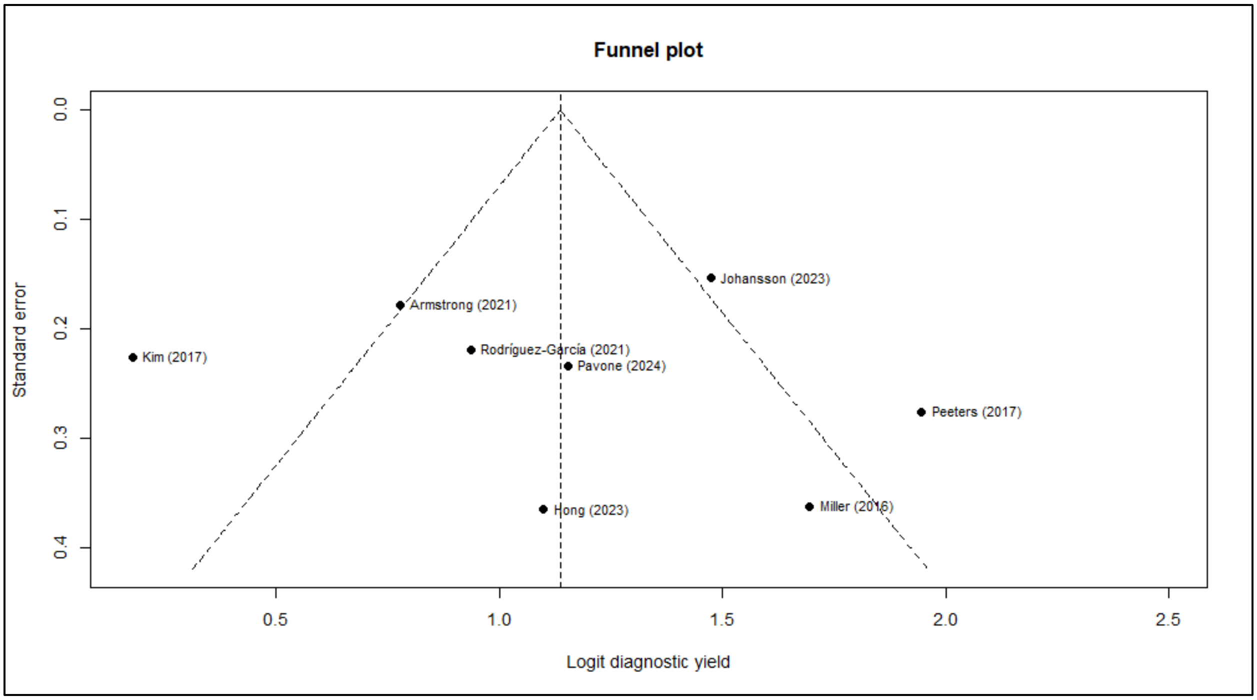

Figure S3. Funnel plot for the primary diagnostic-yield meta-analysis. The plot provides an exploratory graphical assessment of potential small-study effects among the eight studies included in the primary quantitative synthesis. Formal assessments using Egger's regression, Begg-Mazumdar rank correlation, and Peters' regression are reported in Table S9. Because fewer than 10 studies were available, funnel-plot interpretation and formal asymmetry tests were considered exploratory and underpowered; apparent symmetry or non-significant tests should not be interpreted as evidence excluding publication bias.

Table S9. Exploratory tests for funnel-plot asymmetry and small-study effects.

Test	Statistic	df	P value
**Egger regression**	0.288	6	0.783
**Begg rank correlation**	0.495	—	0.621
**Peters regression**	−0.645	6	0.543

Abbreviations: df, degrees of freedom.
Notes: Small-study effects were explored using Egger's regression test, the Begg-Mazumdar rank-correlation test, and Peters' regression test. These tests evaluate different forms of association between study size or precision and observed effect estimates and should not be regarded as direct tests for publication bias. Because only eight studies contributed to the primary meta-analysis, all funnel-plot and regression-based assessments were considered exploratory and statistically underpowered. Accordingly, non-significant test results were interpreted as absence of statistical evidence of funnel-plot asymmetry rather than evidence that publication bias was absent.

Table S10. Feasibility assessment of candidate study-level moderators for subgroup analysis or meta-regression.

Candidate moderator	Distribution	Decision
**Country**	8 countries / 8 studies	Not analyzed; no replication
**Study design**	5 categories	Not analyzed; sparse groups

**Analysis unit**	4 categories	Not analyzed; sparse groups
**PCR target**	4 categories	Not analyzed; sparse groups
**Sequencing method**	4 categories	Not analyzed; sparse groups
**Center structure**	7 single-center, 1 healthcare-zone	Not analyzed; insufficient comparator group
**Availability of accuracy data**	6 no / 2 yes	Not a biologically or methodologically valid moderator
**Valve type**	One study provided extractable native- and prosthetic-valve molecular-positive counts; one reported a qualitative comparison without stratum-specific counts	Descriptive comparison only; insufficient data for subgroup meta-analysis or meta-regression
**Specimen preservation**	One study provided separate molecular-positive counts for fresh and FFPE valve tissue; only five FFPE specimens were included	Descriptive comparison only; insufficient data for subgroup meta-analysis or meta-regression

Abbreviations: NGS, next-generation sequencing; PCR, polymerase chain reaction. **Notes:** Candidate moderators were evaluated before formal subgroup analysis or meta-regression according to clinical relevance, methodological interpretability, and replication across studies. No formal moderator analysis was undertaken when categories contained only one or very few studies, when a variable was excessively fragmented across the eight primary studies, or when the variable represented outcome availability rather than a meaningful clinical or methodological characteristic. The decision not to perform meta-regression was therefore based on insufficient study-level replication and the risk of unstable or non-informative estimates rather than on the absence of potential sources of heterogeneity.

Table S11. Study-level definition of molecular positivity, handling of potentially ambiguous results, and documented non-index microbiological work-up in the primary meta-analysis.

Study	Primary count	Results included in the primary numerator	Treatment of potentially ambiguous findings	Documented non-index microbiological work-up
Miller et al. (2016) [4]	49/58	Study-reported positive 16S rRNA gene PCR/sequencing results among patients with definite or possible IE.	Three culture-negative patients initially classified as possible IE had positive molecular results and were accepted as IE after clinical review. Patients with rejected IE were not included in the primary denominator. Mixed or inconclusive identifications were not independently re-adjudicated by the review authors.	Blood cultures and Gram staining and culture of excised valve tissue were documented. Valve processing included aerobic and anaerobic bacterial culture, and fungal culture was performed routinely. Systematic serology or organism-specific molecular testing was not described.

Kim et al. (2017) [5]	43/79	Study-reported positive 16S rRNA gene PCR/sequencing results among 65 definite and 14 possible IE episodes.	Species- and genus-level identifications reported as positive were retained. Discordant results were interpreted by the original investigators using the other microbiological findings and the clinical context. No molecular-positive result was explicitly excluded as contamination. The abstract-reported numerator of 40 was examined separately in sensitivity analysis.	The documented microbiological comparison comprised blood cultures and bacterial culture of excised valve tissue. Systematic serology or organism-specific molecular assays were not reported.
Peeters et al. (2017) [12]	105/120	Positive valve-tissue 16S rRNA gene PCR/sequencing results in the postoperative definite-IE subgroup.	The publication did not provide a separate patient-level count of molecular-positive results excluded as contaminants or clinically irrelevant. The study-reported positive count was therefore retained without additional retrospective adjudication.	Blood cultures and valve cultures were compared with valve-tissue molecular testing. Serological testing was included in the institutional microbiological protocols referenced by the study, but its systematic application and patient-level results were not fully reported in the main article.
Armstrong et al. (2021) [6]	100/146	All study-reported positive valve-tissue 16S rRNA gene PCR results among cases of histopathologically or macroscopically confirmed active IE.	One result containing eubacterial DNA without successful species identification was included in the study-reported positive count but was not considered an incremental etiologic identification. Skin commensals were considered diagnostically informative when confirmed by concordant valve PCR; discordant results were interpreted in relation to pathogen plausibility.	Blood cultures and valve cultures constituted the routine microbiological comparison. Serology was performed in BCNE or when indicated by patient characteristics, while organism-specific PCR was used selectively when a particular pathogen was suspected. Results from organism-specific PCR assays were not included in the study's principal comparative analysis.
Rodríguez-García et al. (2021) [13]	74/103	All study-reported positive valve-tissue 16S rRNA gene PCR results in the tested surgical IE subgroup.	Genus-level identifications were retained when reported as positive. Molecular findings discordant with culture were interpreted by the original investigators as possible polymicrobial	Blood cultures and bacterial and fungal cultures of excised valve or other cardiac tissue were documented. Systematic serology or organism-specific molecular testing was not

			infection when both organisms were plausible. Reported contaminations predominantly concerned valve cultures rather than molecular results.	described in the reported comparative evaluation.
Hong et al. (2023) [7]	30/40	Study-reported positive 16S rRNA gene PCR/sequencing results among patients classified as having active IE.	Patients without active IE were excluded from the primary denominator. No molecular-positive results were explicitly reported as contaminants or clinically irrelevant within the active-IE subgroup.	Blood cultures, valve cultures, and histopathological findings were documented. Serological and other organism-specific investigations were used selectively in individual BCNE cases, including Bartonella serology and Histoplasma testing; a uniform comprehensive microbiological panel was not reported for all patients.
Johansson et al. (2023) [8]	227/279	16S rRNA gene PCR/sequencing findings classified by the original investigators as possible causative agents.	Two molecular results classified as contaminants according to the study's predefined comparison with blood and valve cultures were excluded. One finding potentially attributable to an IE episode three years earlier was retained by the original investigators, who acknowledged persistent bacterial DNA as an alternative explanation.	The documented microbiological comparison comprised blood cultures and valve cultures. Serology and organism-specific molecular assays were discussed as possible investigations in BCNE, but their systematic use across the cohort was not reported.
Pavone et al. (2024) [11]	76/100	All study-reported positive 16S rRNA gene PCR results in patients undergoing surgery for definite or suspected IE.	No separate molecular-positive findings were explicitly excluded as contaminants or clinically irrelevant. The authors acknowledged that positivity after antimicrobial therapy may represent residual, nonviable bacterial DNA; therefore, these results were retained as molecular detections and were not automatically interpreted as evidence of active infection.	Blood cultures and cultures of excised valve tissue constituted the documented microbiological comparison, with histopathological examination also used in clinical classification. Systematic serology or organism-specific molecular assays were not described.

Abbreviations: BCNE, blood-culture-negative infective endocarditis; IE, infective endocarditis; PCR, polymerase chain reaction. The primary outcome represents study-reported molecular detection. Etiological novelty and clinical benefit were assessed separately through the strict incremental etiologic yield and broad diagnostic-benefit outcomes. The non-index microbiological work-up reflects the investigations documented in each original report. "Not described" does not establish that an investigation was not performed. The included studies did not consistently report a standardized diagnostic algorithm or confirm that every patient underwent the same combination of culture, serology, and organism-specific molecular testing. Consequently, incremental yield was evaluated relative to the documented study-specific microbiological work-up.

Table S12. Detailed laboratory characteristics of valve-tissue 16S PCR and sequencing methods used in studies included in the primary meta-analysis.

Study	Targeted region and primers	Sequencing method and platform	Sequence-quality and similarity criteria	Handling of mixed sequences	Identification database
Miller et al. (2016) [4]	Approximately 500–600 bp covering V1–V3. Dual-priming oligonucleotides: 16SDPO_F, 5′-AGAGTTTGATCMTGGCTCA-I-I-I-I-I-AACGCT-3′, and 16SDPO_R, 5′-CGCGGCTGCTGGCA-I-I-I-A-I-TTRGC-3′.	Sanger sequencing using BigDye Terminator v1.1 on an ABI Prism 3130xl genetic analyzer.	Species- or genus-level identification according to CLSI MM18 identity criteria; numerical thresholds were not reported.	Some results were reported as "mixed"; the method used to resolve mixed chromatograms was not described.	SmartGene IDNS Bacteria database using BLAST.
Kim et al. (2017) [5]	Two primer pairs: 8FPL, 5′-AGTTTGATCCTGGCTCAG-3′, with 806R, 5′-GGACTACCAGGGTATCTAAT-3′; and 515FPL, 5′-TGCCAGCAGCCGCGGTAA-3′, with 13B, 5′-AGGCCCGGGAACGTATTCAC-3′.	Direct Sanger sequencing using BigDye Terminator chemistry and capillary electrophoresis on an ABI Prism 3730xl genetic analyzer.	Sequence quality control and bacterial identification followed CLSI MM18-A; numerical similarity thresholds were not reported.	Cloning was not performed; consequently, the assay was considered unable to reliably detect polymicrobial infections.	GenBank and EzTaxon-e.
Peeters et al. (2017) [12]	Broad-range bacterial 16S assay using the UMD Universal kit (Molzym). The kit's paired amplification primers were proprietary; sequencing primers SeqGN16 and SeqGP16 were used. The precise variable region was not reported.	Sanger amplicon sequencing using BigDye Terminator v3.1 on an ABI Prism 3730	Similarity thresholds of ≥97% for genus-level and ≥99% for species-level identification.	Specific procedures for resolving mixed sequences were not reported.	GenBank using BLAST and the SepsiTest BLAST database.

		DNA analyzer.			
Armstrong et al. (2021) [6]	Primers 357f, 5′-CCTACGGGAGGCAGCAG-3′, and 519r, 5′-ATTACCGCGGCKGCTGG-3′, targeting an approximately 160-bp portion of the 16S gene.	Sequencing of the amplification product; sequencing chemistry and platform were not reported.	Sequence-quality criteria and numerical similarity thresholds were not reported.	No specific method for resolving mixed sequences was reported. One sample contained eubacterial DNA, but sequencing did not provide species-level identification.	BIBI database.
Rodríguez-García et al. (2021) [13]	Broad-range 16S rDNA PCR performed according to a previously published protocol. Primer sequences and the targeted variable region were not restated in the study report.	Sequencing was performed, but the sequencing chemistry and platform were not reported.	Sequence-quality criteria and numerical similarity thresholds were not reported.	Laboratory processing of mixed chromatograms was not described. Clinically discordant findings were assessed using the other microbiological findings and the clinical context.	NCBI BLAST and leBIBI, version 1.1.
Hong et al. (2023) [7]	V1–V3 region amplified using dual-priming oligonucleotides. Primer sequences were not reported in the study article.	Sanger sequencing and/or targeted NGS on an Illumina MiSeq, selected according to an algorithm incorporating PCR cycle-threshold and melting-	Samples with Ct <32 underwent Sanger sequencing. Uninterpretable Sanger results and samples with Ct 32–34 underwent NGS. Samples with Ct >34 underwent NGS only	Sanger sequencing was acknowledged as unable to resolve mixed sequences; NGS was used for samples with uninterpretable Sanger results. All IE cases in the analyzed	Pathogenomix bioinformatics software; the underlying reference database was not specified.

		temperature values.	when the melting-curve peak was ≥0.4. Numerical taxonomic-similarity thresholds were not reported.	cohort were reported as monomicrobial.	
Johansson et al. (2023) [8]	Primers P5f, 5′-TGCCAGCMGCCGCGGTWAT-3′, and P1067r, 5′-ACCATYTCACRACACGAGCT-3′, generating an approximately 570-bp amplicon.	Sanger sequencing; the sequencing instrument was not reported.	Sequence-quality criteria and numerical taxonomic-similarity thresholds were not reported.	Mixed chromatograms were analyzed using RipSeq Mixed Sanger Poly software.	NCBI Nucleotide BLAST database.
Pavone et al. (2024) [11]	Broad-range primers targeting the bacterial 16S rRNA gene, supplemented by organism-specific primers for *Tropheryma whipplei*, *Coxiella burnetii*, and *Bartonella* species. Primer sequences and the targeted 16S variable region were not reported.	Sequencing of amplified DNA was performed; sequencing chemistry and platform were not reported.	Sequence-quality criteria and numerical similarity thresholds were not reported.	Handling of mixed sequences was not reported.	The authors reported comparison with established databases but did not specify their names.

Abbreviations: BLAST, Basic Local Alignment Search Tool; CLSI, Clinical and Laboratory Standards Institute; Ct, cycle threshold; IE, infective endocarditis; NGS, next-generation sequencing; NR, not reported; PCR, polymerase chain reaction. I denotes deoxyinosine in the dual-priming oligonucleotide sequences. Laboratory information was extracted from the primary reports and, when explicitly referenced, their associated published assay protocols. NR indicates that the corresponding information could not be verified from the published material.

Table S13. Study-reported organism-specific findings from valve-tissue 16S rRNA gene PCR and sequencing in studies included in the primary meta-analysis.

Study	Molecular-only or additional etiological identifications	Fastidious, intracellular, or otherwise notable organisms detected molecularly	Concordance with conventional microbiology	Discordant findings, possible contaminants, and interpretive considerations
Miller et al. (2016) [4]	New molecular diagnoses in definite IE included *Streptococcus dysgalactiae*, *Staphylococcus lugdunensis*, and *Cutibacterium acnes*	Molecular testing also identified organisms that may be difficult to recover after antibiotic exposure, including	In definite IE, molecular findings agreed with blood or	Molecular testing clarified several culture results, including a case with coagulase-negative

	(reported as *Propionibacterium acnes*). Three culture-negative possible-IE cases had molecular detection of *Acinetobacter junii*, *Delftia tsuruhatensis*, and a viridans-group streptococcus and were accepted as IE after clinical review.	*Granulicatella adiacens*, *Haemophilus parainfluenzae*, *Cardiobacterium hominis*, and *C. acnes*.	valve culture in 37/50 cases (74%).	staphylococci in blood culture but *Enterococcus faecalis* by valve PCR. One *E. faecalis* molecular result in a patient with rejected IE and mixed molecular signals in non-IE cases were excluded from the primary IE numerator.
Kim et al. (2017) [5]	Eight cases were positive only by molecular testing: *Streptococcus constellatus*, *Streptococcus sanguinis*, *Streptococcus salivarius*, *Haemophilus parainfluenzae*, *Granulicatella adiacens*, *Aggregatibacter actinomycetemcomitans*, *Abiotrophia* spp., and *Propionibacterium humerusii*.	The molecular-only findings included nutritionally variant streptococci, *H. parainfluenzae*, and *A. actinomycetemcomitans*.	Molecular findings were accompanied by concordant blood culture in 18 cases, concordant valve culture in 2 cases, and both culture methods in 15 cases.	Three discordant cases were reported. Valve cultures yielding *Staphylococcus epidermidis* and *Klebsiella pneumoniae* were considered contaminants when blood culture and molecular testing agreed on *E. faecalis* and *S. sanguinis*, respectively. A blood-culture finding of *Actinomyces* spp. and molecular identification of *Streptococcus mutans* were interpreted as possible coinfection.
Peeters et al. (2017) [12]	Nine molecular-only etiological identifications were reported. Among PCR-positive BCNE cases, detected organisms included *Cutibacterium acnes* (reported as *Propionibacterium acnes*), coagulase-negative staphylococci, *Streptococcus gallolyticus*, *Streptococcus mitis*, *Coxiella burnetii*, *Aggregatibacter actinomycetemcomitans*, and	Clinically notable molecular detections included *C. burnetii*, *A. actinomycetemcomitans*, *Abiotrophia defectiva*, *Granulicatella adiacens*, and *H. parainfluenzae*.	Molecular findings confirmed or agreed with a substantial proportion of culture-based etiological diagnoses, although a complete patient-level concordance count was not	Molecular testing clarified mixed or discordant culture findings in 11 cases. The publication did not provide a separate count of molecular-positive results classified as contaminants. One complex discordant case involved blood culture positive for *Pseudomonas aeruginosa*, valve

	a mixed *Streptococcus bovis/Staphylococcus* finding.		provided in the main report.	culture positive for *P. aeruginosa* and *Streptococcus sanguinis*, and molecular detection of *E. faecalis* and *Acinetobacter* spp.
Armstrong et al. (2021) [6]	Valve PCR was the only positive microbiological method in 19 cases, identifying *Abiotrophia defectiva*, *Aggregatibacter actinomycetemcomitans*, *Corynebacterium kroppenstedtii*, *Coxiella burnetii*, *Enterococcus faecalis*, *Klebsiella oxytoca*, *Staphylococcus epidermidis*, *Staphylococcus haemolyticus*, *Streptococcus agalactiae*, *Streptococcus dysgalactiae*, *Streptococcus gordonii*, *Streptococcus milleri*, *Streptococcus mitis*, *Streptococcus pasteurianus*, *Streptococcus salivarius*, and *Tropheryma whipplei*.	The cohort included two molecular detections each of *C. burnetii* and *T. whipplei*, subsequently supported by serology and organism-specific valve PCR, respectively.	Among 76 patients with positive blood culture and valve PCR, 63 had concordant species. Molecular testing also confirmed a blood-culture skin commensal as the likely pathogen in 11 cases.	Seven blood culture–PCR species discordances were reported. In four, molecular testing replaced a blood-culture skin commensal with a more plausible pathogen: *T. whipplei*, *C. burnetii*, *Staphylococcus aureus*, or *Streptococcus mitis/oralis*. Six comparisons were inconclusive, including one result with eubacterial DNA but no species identification.
Rodríguez-García et al. (2021) [13]	Sixteen cases with negative blood and valve cultures were positive only by molecular testing: *Coxiella burnetii* (n=4), *Tropheryma whipplei* (n=2), *Streptococcus mitis* (n=2), and one each of *Staphylococcus aureus*, *Staphylococcus epidermidis*, *Escherichia coli*, *Staphylococcus lugdunensis*, *Streptococcus agalactiae*, *Bartonella henselae*, *Streptococcus mutans*, and *Chryseobacterium* spp.	The molecular-only findings included *C. burnetii*, *T. whipplei*, and *B. henselae*.	Blood-culture and molecular identifications were concordant in all but two cases in which both methods were positive. Molecular testing also supported the etiological relevance of several skin-commensal culture findings.	Two blood culture–PCR discrepancies were interpreted as possible polymicrobial infections: *Candida albicans* with *S. dysgalactiae*, and *Granulicatella adiacens* with *S. epidermidis*. Reported contaminations predominantly involved valve culture rather than molecular testing.
Hong et al. (2023) [7]	Among 11 molecular-positive BCNE cases, five detections lacked culture or serological corroboration:	Three cases of *Bartonella* spp. were detected molecularly and supported by high	Three of the 11 molecular-positive BCNE cases had the	Two culture–molecular discrepancies were described: valve-

	Cutibacterium acnes (n=2), *Haemophilus parainfluenzae*, *Streptococcus mitis* group, and *Streptococcus sanguinis*.	*Bartonella henselae* IgG titres. *H. parainfluenzae* was also identified.	same organism identified by valve culture: *C. acnes* in two cases and *S. epidermidis* in one. The three *Bartonella* detections were supported serologically.	culture *C. acnes* versus molecular *Bartonella* spp. supported by serology, and valve-culture *Staphylococcus hominis*/*S. epidermidis* versus molecular *C. acnes*. The etiological relevance of *C. acnes* and coagulase-negative staphylococci required clinical interpretation. A case of *Histoplasma* endocarditis diagnosed by serology and histopathology was not detected by the bacterial 16S assay.
Johansson et al. (2023) [8]	Molecular testing provided an etiological finding in 15/20 BCNE episodes. The study specifically highlighted two *Tropheryma whipplei* detections and one *Streptococcus bovis* finding in a patient with previous *S. bovis* IE.	Molecular findings included *T. whipplei*, *Abiotrophia defectiva*, *Granulicatella* spp., *Capnocytophaga canimorsus*, *Aerococcus urinae*, *Rothia* spp., and HACEK-group organisms.	Molecular results were concordant with blood cultures in 214/279 episodes (77%). The most frequent molecular identifications were *S. aureus*, viridans-group streptococci, and enterococci.	Two molecular results were classified as contaminants and excluded by the original investigators. The *S. bovis* finding could represent persistent DNA from an IE episode three years earlier rather than the current infection. Environmental organisms, *C. acnes*, and polymicrobial findings were considered in the study-specific contamination assessment.
Pavone et al. (2024) [11]	Seven cases were positive only by molecular testing: *Streptococcus agalactiae* (n=2), *Streptococcus oralis*,	Clinically notable molecular-only findings included *C. burnetii* and	Among 64 patients with positive blood culture and	Molecular testing contributed to the interpretation of four cases with discordant

	Streptococcus tigurinus, *Enterococcus faecalis*, *Coxiella burnetii*, and *Fusobacterium nucleatum*.	the anaerobic organism *F. nucleatum*.	molecular testing, 54 (84%) had concordant organisms. Among 43 patients with positive valve culture and molecular testing, 39 (90%) were concordant.	blood and valve cultures. The authors emphasized that organisms such as *S. epidermidis* and *S. cohnii* may represent contaminants and that discordant results require case-by-case assessment using the third microbiological test and the clinical context.

Abbreviations: BCNE, blood-culture-negative infective endocarditis; CoNS, coagulase-negative staphylococci; IE, infective endocarditis; PCR, polymerase chain reaction. Organism-level findings were extracted according to the classifications and terminology of the original studies. "Molecular-only" indicates that valve-tissue 16S rRNA gene PCR/sequencing was the only positive microbiological method within the comparison reported by the respective study; the extent of serological and organism-specific testing differed across studies. Lists are exhaustive only when complete patient-level information was provided by the original publication. Because reporting formats and definitions of concordance, discordance, and contamination differed, organism-specific findings were summarized descriptively and were not quantitatively pooled. Species-level labels reproduce the identifications reported by the original investigators and do not imply equivalent or independently validated taxonomic resolution across assays.
